# A Neuro-hormonal Circuit for Paternal Behavior Controlled by a Hypothalamic Network Oscillation

**DOI:** 10.1016/j.cell.2020.07.007

**Published:** 2020-08-20

**Authors:** Stefanos Stagkourakis, Kristina O. Smiley, Paul Williams, Sarah Kakadellis, Katharina Ziegler, Joanne Bakker, Rosemary S.E. Brown, Tibor Harkany, David R. Grattan, Christian Broberger

**Affiliations:** 1 Department of Neuroscience, Biomedicum B4, Karolinska Institutet, Solnavägen 9, 171 65 Stockholm, Sweden; 2 Centre for Neuroendocrinology, School of Biomedical Sciences, University of Otago, Dunedin 9016, New Zealand; 3 Department of Anatomy, School of Biomedical Sciences, University of Otago, Dunedin 9016, New Zealand; 4 Department of Biochemistry and Biophysics, Stockholm University, Svante Arrhenius väg 16C, 106 91 Stockholm, Sweden; 5 Department of Physiology, School of Biomedical Sciences, University of Otago, Dunedin 9016, New Zealand; 6 Department of Molecular Neurosciences, Center for Brain Research, Medical University of Vienna, Spitalgasse 4, 1090 Vienna, Austria; 7 Maurice Wilkins Centre, University of Auckland, Auckland, New Zealand

**Keywords:** arcuate nucleus, dopamine, gap junction, medial preoptic area, neuronal network, oscillation, parental, paternal, prolactin, species difference

## Abstract

Parental behavior is pervasive throughout the animal kingdom and essential for species survival. However, the relative contribution of the father to offspring care differs markedly across animals, even between related species. The mechanisms that organize and control paternal behavior remain poorly understood. Using Sprague-Dawley rats and C57BL/6 mice, two species at opposite ends of the paternal spectrum, we identified that distinct electrical oscillation patterns in neuroendocrine dopamine neurons link to a chain of low dopamine release, high circulating prolactin, prolactin receptor-dependent activation of medial preoptic area galanin neurons, and paternal care behavior in male mice. In rats, the same parameters exhibit inverse profiles. Optogenetic manipulation of these rhythms in mice dramatically shifted serum prolactin and paternal behavior, whereas injecting prolactin into non-paternal rat sires triggered expression of parental care. These findings identify a frequency-tuned brain-endocrine-brain circuit that can act as a gain control system determining a species’ parental strategy.

## Highlights

• Species-specific hypothalamic dopamine neuron rhythms yield distinct prolactin release
• Serum prolactin primes the “parental” neural circuit for pup care during fatherhood
• Optogenetic control of TIDA frequency tunes prolactin and paternal behavior
• Prolactin receptors in the MPOA are required for paternal behavior

## Graphical Abstract

**Figure:**
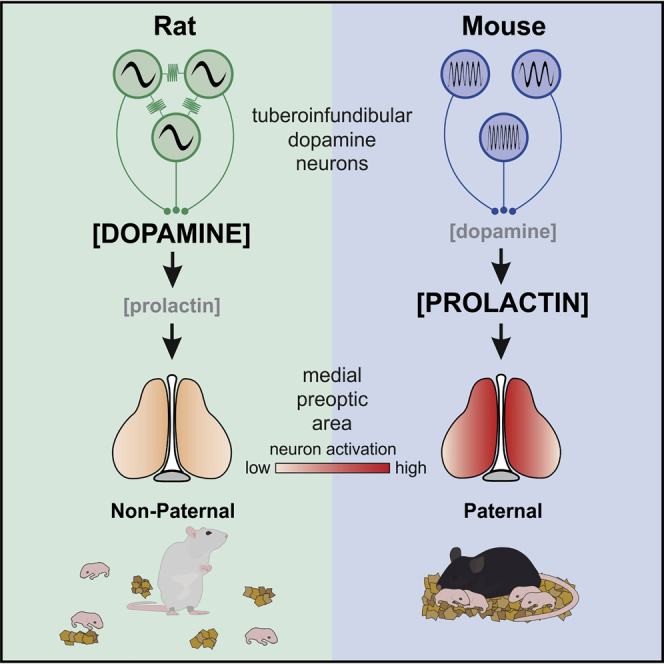

## Teaser

Parenting strategies in males are determined by species-specific oscillation frequencies in hypothalamic dopamine neurons that define circulating prolactin levels and baseline activity of the parental neural circuit.

## Introduction

Parental strategies shape society because they directly affect the physical and mental well-being of the young generation and emergence of their social skills (Feldman, 2015; Galende et al., 2011; Scott and Jean-Baptiste, 2012; van der Pol et al., 2016). A parent’s attention influences the development of a child’s cognitive functions (Ingram and Ritter, 2000; Cox et al., 2003), and neglect or abuse can predispose them to depression (McLeod, 1991; Ross and Mirowsky, 1999), hostility, criminal behavior (Mäki et al., 2003; Moffitt, 1987), and psychiatric illness in adulthood (Yoo et al., 2006; Gregory, 1958).

From an evolutionary perspective, parental behavior is essential for ensuring the survival of a species (van der Pol et al., 2016; Feldman, 2015; Scott and Jean-Baptiste, 2012; Galende et al., 2011) and manifests in different forms throughout the animal kingdom (Ketterson and Nolan, 1994; Greenberg, 1961; Zeveloff and Boyce, 1980). From insects to mammals, the neural substrates encoding this behavior, which has evolved repeatedly across vertebrate and invertebrate taxa, are under strong evolutionary pressure (Mcnamara and Wolf, 2015; Clutton-Brock, 1991; Geary and Flinn, 2001). This has shaped the expression of parental behavior based on its adaptive value in each species, ranging from egg-laying site selection or brooding to provisioning, nursing, and teaching of skills.

Many mammalian species are maternally uniparental, but there are exceptions (Feldman, 2015; Rilling, 2013; Dulac et al., 2014). Classic examples of the biparental approach are the prairie vole (Oliveras and Novak, 1986) and California mouse (Horner, 1947; Dudley, 1974), species in which the males express similar levels of parental care as the lactating females. Interestingly, closely related species can fall at opposite ends of the biparental spectrum (Oliveras and Novak, 1986; Bendesky et al., 2017), indicative of the dynamic range of the neural and endocrine mechanisms underlying this behavior across the phylogenetic tree.

Maternal behavior is strongly influenced by hormones. A seminal study by Terkel and Rosenblatt (1968) revealed that transfusion of blood plasma from a lactating dam can induce maternal behavior in virgin female rats. Subsequent work identified that a triad of hormones (estrogen, progesterone, and prolactin [Prl]), whose levels rise and remain elevated in the rodent female throughout pregnancy, postpartum, and during raising of the young, are responsible for induction of maternal behavior (Moltz et al., 1970). Along with these hormones, oxytocin has been found to play a crucial role in induction and regulation of maternal behavior (Pedersen and Prange, 1979; Keverne et al., 1988). An imposing catalog of later studies has further fortified the association between maternal hormones and maternal behavior (Zarrow et al., 1971; Gonzalez and Deis, 1986).

The hormone most strongly and specifically implicated in maternal functions is Prl, whose secretion from the anterior pituitary is primarily controlled by tuberoinfundibular dopamine (TIDA) neurons located in the dorsomedial arcuate nucleus (dmArc) of the hypothalamus, which exert a powerful inhibitory influence under most physiological conditions in males and virgin females (Gudelsky, 1981; Demarest et al., 1986). This dopamine-mediated inhibition of Prl release operates via activation of the D2 dopamine receptor on Prl-producing lactotroph cells (Gonzalez-Iglesias et al., 2008). Plastic changes in the lactotropic axis are believed to cause the dopaminergic “brake” to lift, resulting in the surge of serum Prl occurring during the late stages of pregnancy and postpartum (Wang et al., 1993; Demarest et al., 1983). This sustained elevation of Prl in the lactating dam is widely recognized to facilitate the expression of lactation and maternal behaviors necessary for raising the offspring (Bridges et al., 1974; Korányi et al., 1977; Brown et al., 2017).

However, although a rich and growing literature has shed light on neural and hormonal regulation of maternal behavior, the mechanisms underlying paternal care for offspring in biparental species remain obscure. It is further not known whether the prerequisites for paternal behavior are in place by default or induced by the process of fathering offspring, as is the case for infanticidal instincts, which are present in the virgin male but largely disappear after mating (vom Saal, 1985). Curiously, the male sire in a biparental species is less prone to somatosensory signals from the young and does not respond by increasing serum Prl to levels found in the lactating dam (Fleming et al., 2002; Guillou et al., 2015; Bridges, 1983). These observations raise the question of whether paternal behavior is a predisposed state rather than induced via somatosensory input.

The current study was motivated by the recent demonstration of differential activity of TIDA neurons in the males of two commonly used animal models, the rat and the mouse (Stagkourakis et al., 2018). The cellular mechanism of this discrepancy was identified as the presence of strong gap junction coupling between male rat TIDA neurons, contrasting with the complete absence of electrical connectivity in male mice (Stagkourakis et al., 2018). These observations at the network level beg the question of the potential functional effect of species differences in TIDA oscillation frequency. The role of Prl, the downstream target of TIDA activity, is poorly understood in males, in particular how it pertains to behavior and parenting. Curiously, mating experience allows the behavioral transition from infanticide to paternal behavior, although only in biparental species (McCarthy and Vom Saal, 1986; Voloschin et al., 1998). Thus, filling this gap in knowledge requires not only identifying the effect of Prl on male action patterns but also determining under which conditions Prl may affect male behavior toward offspring: is it necessary, sufficient, and/or permissive?

We hypothesized that the species difference in TIDA circuit configuration may affect one of the characteristics most strongly associated with Prl in the female: care for offspring. We explored this hypothesis, providing a link between the individual components of the lactotropic axis and the central circuits associated with parental behavior. The correlations we found were then evaluated for causality by manipulating the system in mice with optogenetics and conditional deletion of the Prl receptor (Prl-R) and in rats through pharmacology.

The results demonstrate that the distinct oscillatory activity of TIDA neurons establishes the level of serum Prl throughout adulthood, regulating the activity of neural networks implicated in parental behavior and ultimately enabling (or not) the expression of paternal care. This work provides a mechanistic and conceptual understanding of the role of the lactotropic axis in male parental behavior. It further suggests that pre-set hypothalamic neuron activity at the level of network oscillation frequency and serum Prl levels, prime the conditional expression of a behavioral repertoire that can be elicited in the presence of a sire’s own pups.

## Results

### TIDA Neuron Activity Correlates with Dopamine Release and Serum Prl

**Figure 1:**
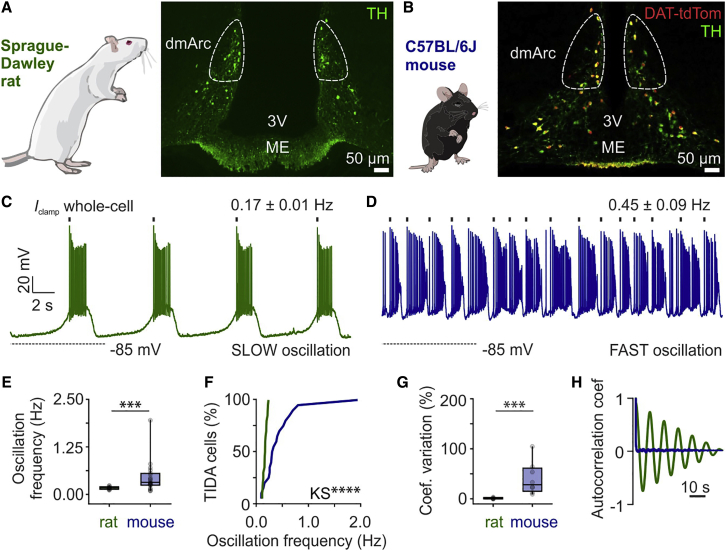
Different Oscillation Frequencies of TIDA Neurons in Male Rats and Mice (A and B) Tuberoinfundibular dopamine (TIDA) neurons in the dorsomedial arcuate nucleus (dmArc) in the male Sprague-Dawley rat (A) and C57BL/6J mouse (B), as visualized by immunofluorescence for tyrosine hydroxylase (TH; rat), and transgenic expression of tdTomato under control of the dopamine transporter promoter (DAT-tdTom; mouse), respectively. A near total colocalization between DAT-tdTom and TH is seen in the dmArc (B). ME, median eminence; 3V, third ventricle. (C and D) *In vitro* whole-cell patch-clamp recordings from rat (C) and mouse (D) TIDA cells show slow and fast oscillatory activity, respectively (n = 20 neurons from 10 animals per species). (E) Quantification of oscillation frequency in rat and mouse TIDA neurons (n = 20 per group, two-sided Mann-Whitney *U* test, p = 0.0002). (F) Exact cumulative distribution of oscillation frequencies recorded in rat (green) and mouse (blue) TIDA neurons (n = 20 per group, Kolmogorov-Smirnov test with Bonferroni correction, p < 0.0001). (G) Coefficient of variation of membrane potential among TIDA neurons recorded in the same slice in rat and mouse (n = 6–8 slices per group, two-sided Mann-Whitney *U* test, p = 0.0007). (H) Representative autocorrelation coefficient plots from rat (green) and mouse (blue) TIDA neurons, indicative of regular and irregular oscillatory activity, respectively. ns, not significant; ^∗∗∗^p < 0.001. In box-and-whisker plots, center lines indicate medians, box edges represent the interquartile range, and whiskers extend to the minimal and maximal values. All experiments were performed in male rodents.

Recent studies have described a striking species difference in the rhythmic electrical activity of Prl-inhibiting TIDA neurons in the male rat and mouse (Lyons et al., 2010; Romanò et al., 2013; Stagkourakis et al., 2018). In agreement with these observations, male rat TIDA neurons were observed to discharge in robust slow oscillations (typically 0.17 Hz; Figures 1A, 1C, 1E). In contrast, male mouse TIDA neurons exhibit significantly faster oscillation frequencies (typically 0.45 Hz; Figures 1B, 1D, and 1E). Notably, male rat TIDA neurons are synchronized and oscillate at the same frequency across cells, slices, and animals, whereas male mouse TIDA neurons are not coordinated and occupy a wide spectrum of faster rhythms (Figures 1F–1H; Stagkourakis et al., 2018).

**Figure 2:**
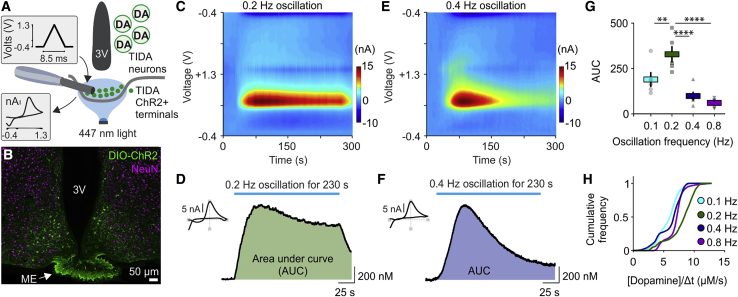
Slow TIDA Neuron Oscillation Can Sustain Dopamine Release, and Faster Oscillations Lead to Dopamine Depletion at the TIDA Terminals (A) Experimental setup to determine the relationship between TIDA oscillation frequency and dopamine release. Brain slices from mice expressing channelrhodopsin (ChR2) in TIDA neurons were exposed to photostimulation of different frequencies, and dopamine concentrations were recorded in the ME by FSCV. (B) Hypothalamic arcuate slice with DAT-driven ChR2-eYFP expression, showing TIDA neuron somata in the arcuate nucleus and dense terminals in the ME. Neuronal cell nuclei were visualized by NeuN immunofluorescence (purple). (C and D) Dopamine release recorded in the ME in response to the 0.2-Hz oscillation frequency applied via a photostimulation protocol. Shown are a fast-scan cyclic voltammetry (FSCV) color plot (C), cyclic voltammogram (D, left), and optically evoked [dopamine] versus time plot (D, right). Note the long sustained dopamine release. (E and F) Dopamine release recorded in the ME in response to the 0.4-Hz oscillation frequency applied via a photostimulation protocol. Shown are an FSCV color plot (E), cyclic voltammogram (F, left), and optically evoked [dopamine] versus time plot (F, right). Note the initial peak of dopamine release that decays rapidly. (G) Dopamine concentration (quantified via area under the curve [AUC] as shown in D and F) plotted for the four oscillation frequencies applied via photostimulation (n = 5 per group, one-way ANOVA with Tukey’s test, p = 0.0038 between 0.1 Hz versus 0.2 Hz, p < 0.0001 between 0.2 Hz versus 0.4 Hz, p < 0.0001 between 0.2 Hz versus 0.8 Hz comparison). Dopamine release drops dramatically between 0.2 Hz and 0.4 Hz. (H) Cumulative frequency of dopamine release over time identifies 0.2-Hz stimulation as optimal for maximal output. Kolmogorov-Smirnov test with Bonferroni correction, p < 0.0001 between 0.2 Hz and 0.1 Hz; Kolmogorov-Smirnov test with Bonferroni correction, p < 0.0001 between 0.2 Hz and 0.4 Hz; Kolmogorov-Smirnov test with Bonferroni correction, p < 0.0001 between 0.2 Hz and 0.8 Hz. ^∗∗^p < 0.01, ^∗∗∗^p < 0.001, ^∗∗∗∗^p < 0.0001. In bar graphs, data are expressed as mean ± SEM. All experiments were performed in male rodents.

The difference between male rat versus mouse TIDA neuron rhythms prompted the question of whether and how differences in oscillation frequency are reflected in TIDA output. We addressed this issue by using optogenetic stimulation to impose a range of electrical frequencies on TIDA neurons and recorded the resultant dopamine concentrations at the release site in the median eminence (ME) by fast-scan cyclic voltammetry (FSCV; Figure 2A). Stimulation of axon terminals rather than cell somata was performed because of concerns that the axonal connection may be lost during preparation of the mediobasal hypothalamus slice due to the complex three-dimensional nature of the dmArc and its projections (Voloschin et al., 1998). Dopamine Transporter (DAT)-Cre mice were injected with Cre-dependent channelrhodopsin (ChR2) in the dmArc (Figure 2B), and frequencies spanning 0.1–0.8 Hz were applied. Optogenetic stimulation yielded dose-dependent patterns of dopamine release patterns in the ME. Notably, 0.2 Hz (“rat-like”) and 0.4 Hz (“mouse-like”) stimulation resulted in distinct dopamine release patterns in the ME. When 0.2-Hz stimulation was applied, the dopamine signal in the ME peaked quickly and remained high until light stimulation was terminated (Figures 2C and 2D). In contrast, when the 0.4-Hz protocol was applied, [dopamine] rose with similar kinetics but decayed quickly after the initial peak (Figures 2E and 2F). Comparisons of different stimulation protocols revealed an inverted U-shaped frequency-response relationship peaking at 0.2 Hz (Figures 2G and 2H). The FSCV experiments suggest that the different electrophysiology patterns found in rat and mouse TIDA neurons lead to sustainable versus non-sustainable dopamine release, respectively, at the TIDA terminals. These findings, in conjunction with the inverse relationship between neuroendocrine dopamine and pituitary Prl, raise the possibility that rat and mouse males may have different serum Prl concentrations.

**Figure 3:**
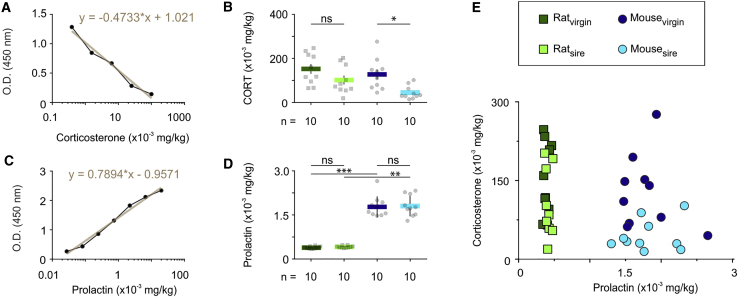
Sexual Experience-Independent and Inverse Serum Prl in Rat and Mouse Males (A) Mean standard curve ranging from 0.3–100 × 10^−3^ mg/kg, used to calculate corticosterone (CORT) levels in male rat and mouse serum samples. The measured optical density (OD) is linearly proportional to [CORT]_serum_. (B) Quantification of CORT levels (n = 10 animals per group, one-way ANOVA with Tukey’s test, p = 0.2663 between virgin and sire rat samples, p = 0.0217 between virgin and sire mouse samples). (C) Mean standard curve ranging from 0.015–20 × 10^−3^ mg/kg, used to calculate Prl levels in male rat and mouse serum samples. (D) Quantification of Prl levels (n = 10 animals per group, Kruskal-Wallis test followed by Dunn’s test, p > 0.9999 between virgin and sire rat samples, p > 0.9999 between virgin and sire mouse samples, p = 0.0002 between virgin rat and virgin mouse samples, p = 0.0032 between rat sire and mouse sire samples). (E) Plot of CORT and Prl levels, showing completely segregated Prl serum concentrations between rat and mouse. ^∗^p < 0.05, ^∗∗^p < 0.01, ^∗∗∗^p < 0.001. In bar graphs, data are expressed as mean ± SEM. All experiments were performed in male rodents.

To test whether this is the case, we collected tail blood samples in male rats and mice and quantified serum [Prl] by ELISA. Each sample was also tested for corticosterone (CORT; Figures 3A, 3B, and 3E) because acute stress brought about by the sampling procedure can influence serum Prl levels (Neill, 1970; Seggie and Brown, 1975). Although rare (n = 5 of 45), samples with [CORT] exceeding 300 × 10^−3^ mg/kg were excluded from serum Prl measurements and further analysis. Standard curves using standardized ELISA protocols for CORT and Prl were used to calculate the absolute levels of these hormones in the serum (Figures 3A and 3C). Rat serum [Prl] was 0.3889 ± 0.0129 × 10^−3^ mg/kg in the virgin male and 0.4150 ± 0.0121 × 10^−3^ mg/kg in the sire (Figures 3D and 3E). In contrast, serum [Prl] in the male mouse was found to be severalfold higher than in the rat (1.784 ± 0.1136 × 10^−3^ mg/kg in virgins and 1.8120 ± 0.1121 × 10^−3^ mg/kg in sires). Importantly, serum [Prl] did not differ between physiological states in the rat or mouse, with virgins and sires having comparable serum Prl concentrations (Figures 3D and 3E).

### Low and High Prl-R Activation in the Male Rat and Mouse MPOA, Respectively

**Figure 4:**
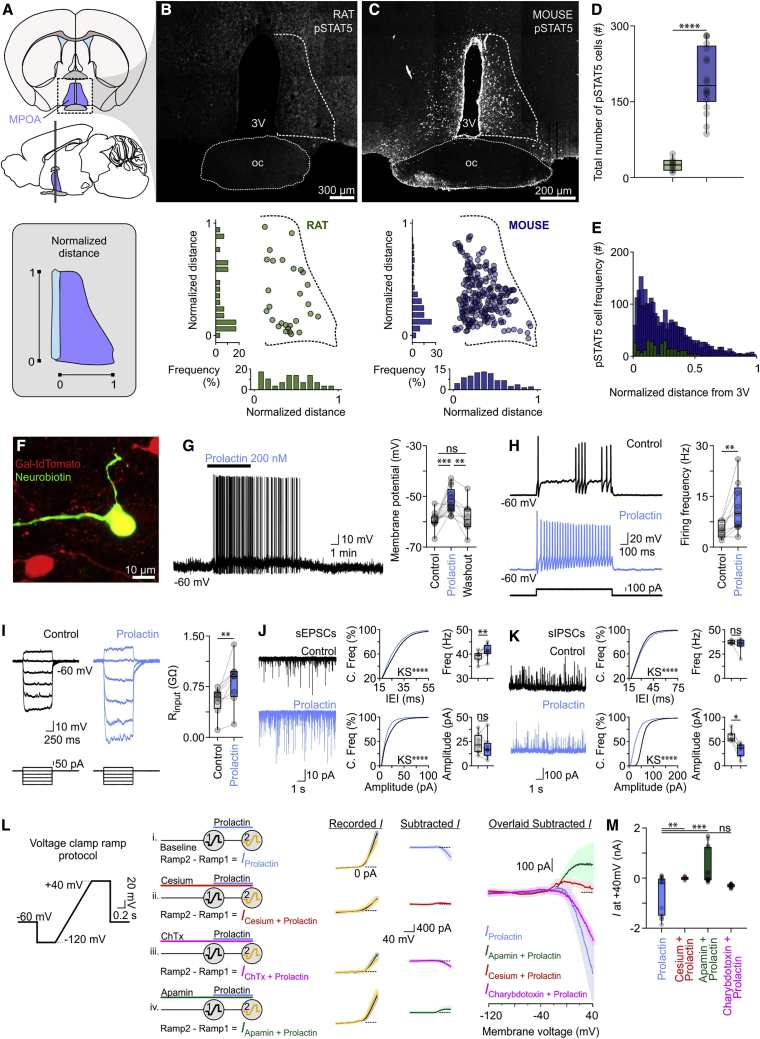
Low and High Activation of Prl-R Signaling in the MPOA of Male Rats and Mice, Respectively, and Depolarization of *Gal*^+^ Neurons by Prl (A) Identification of the MPOA in rat and mouse hypothalamic sections and normalization of the mediolateral and dorsoventral axes for comparison between mouse and rat. (B) Representative confocal image and quantification of immunofluorescence for pSTAT5 (a downstream signal transducer of the Prl-R) in the MPOA in the adult virgin male rat under no manipulation (n = 12). oc, optic chiasm. (C) Representative confocal image and quantification of pSTAT5 immunofluorescence in the MPOA in the adult virgin male mouse under no manipulation (n = 18). (D) Quantification of pSTAT5 cells in the rat (green) and mouse (blue) MPOA (n = 12–18 per group, two-tailed unpaired t test, p < 0.0001). (E) Quantification of pSTAT5 cells with mediolateral distribution in the MPOA (n = 12–18 per group). (F) *Gal*-tdTomato (red) MPOA neuron recorded in whole-cell mode and reconstructed after filling with neurobiotin (green; n = 6). (G) Whole-cell recording of a *Gal*^+^ MPOA neuron shows reversible excitation and induction of firing by bath application of Prl (left). Right: quantification of Prl-induced membrane potential depolarization (n = 13, control versus Prl, paired t test, p = 0.0005; Prl versus washout, paired t test, p = 0.0087). (H) Left: representative response to a depolarizing square pulse in a *Gal*^+^ MPOA neuron under control conditions (top) and in the presence of Prl (bottom). Right: quantification of the firing frequency under control conditions and following application of Prl (n = 9, paired t test, p = 0.0022). (I) Current pulse injection of varying amplitude in a *Gal*^+^ MPOA neuron under control conditions (left) and upon application of Prl (center) yields different membrane voltage responses. Input resistance under control conditions and in the presence of Prl is quantified on the right (n = 9, paired t test, p = 0.0039). The experiment was performed in the presence of tetrodotoxin (500 nM) to isolate postsynaptic actions. (J) Spontaneous excitatory postsynaptic currents (sEPSCs) increase in a *Gal*^+^ neuron upon application of Prl (left). Shown is the cumulative frequency distribution of the sEPSC inter-event interval (IEI) and amplitude (center, n = 8 cells, Kolmogorov-Smirnov test with Bonferroni correction, p < 0.0001 between control and Prl). Also shown is comparison of sEPSC frequency (top right, n = 8, two-tailed paired t test, p = 0.0070) and amplitude (bottom right, n = 8, two-tailed paired t test, p = 0.2250). (K) Spontaneous inhibitory postsynaptic currents (sIPSCs) recorded from a *Gal*^+^ neuron under control conditions and upon application of Prl (left). Cumulative frequency distribution of sIPSC IEI and amplitude (center, n = 7 cells, Kolmogorov-Smirnov test with Bonferroni correction, p < 0.0001 between control and Prl). Also shown is comparison of sIPSC frequency (top right, n = 7, two-tailed paired t test, p = 0.3308) and amplitude (bottom right, n = 7, two-tailed paired t test, p = 0.0125). (L) Schematic illustration of the voltage-clamp ramp protocol used to identify the Prl mediated currents (far left) and a schematic illustration of the experimental designs, i–iv, used to identify Prl-mediated currents and involvement of K^+^ conductances (left; ChTx, charybdotoxin). Also shown is the average with the voltage-clamp ramp recordings under control versus Prl application conditions (center; 8–10 cells per current; control traces are illustrated in black with gray standard error, whereas traces following Prl application are illustrated in orange with light orange standard error), identification of the Prl-mediated current and its modulation by different K^+^ channel blockers via digital subtraction of Ramp2 – Ramp1 (right), and overlaid digitally subtracted current (far right). (M) Quantification of currents at +40 mV (duplicate ramp recording measurements from 8–10 cells; comparisons were performed with Kruskal-Wallis test followed by Dunn’s test, p = 0.0027 between Prl and cesium + Prl, p = 0.0005 between Prl and apamin + Prl, p = 0.0976 between Prl and ChTx + Prl). ^∗^p < 0.05, ^∗∗^p < 0.01, ^∗∗∗^p < 0.001, ^∗∗∗∗^p < 0.0001. In box-and-whisker plots, center lines indicate medians, box edges represent the interquartile range, and whiskers extend to the minimal and maximal values. All experiments were performed in male rodents. See also Figure S1.

Having established a difference in serum Prl in rats and mice, we next addressed whether this discrepancy is reflected in the degree of Prl signaling in the brain. We focused on the medial preoptic area (MPOA; Figure 4A), a region that has been strongly implicated in regulation of parental behavior (Lee and Brown, 2007; Wu et al., 2014; Kohl et al., 2018; Numan et al., 1977). Activation of the Prl-R in the MPOA in lactating dams is necessary for the expression of maternal behavior and survival of the offspring (Brown et al., 2017). Phosphorylation of signal transducer and activator of transcription 5 (STAT5) is a key downstream step of Prl-R activation and provides a reliable readout of Prl signal transduction (Gouilleux et al., 1994; Lerant et al., 2001; Brown et al., 2010). In the male virgin rat, only occasional pSTAT5-immunoreactive cells were observed in the MPOA (Figures 4B, 4D, and 4E), whereas there was intense staining for this activity marker in the male virgin mouse MPOA (Figures 4C–4E), suggesting baseline activation of a key nucleus for organization of parental behavior in the murine brain. We next explored whether MPOA neurons can be stimulated directly by Prl.

### Prl Excites MPOA *Gal*^+^ Neurons

**Figure S1:**
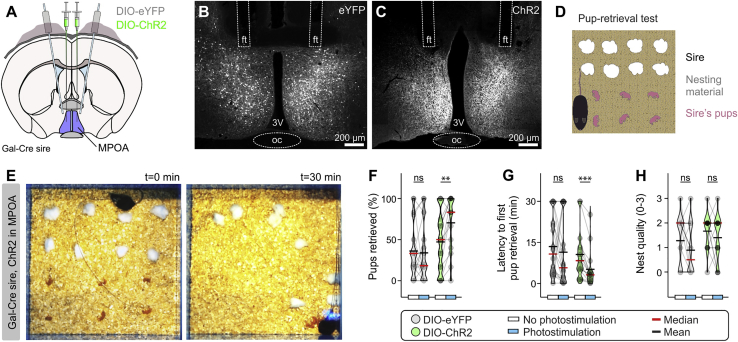
Optogenetic Activation of MPOA *Gal*^+^ Neurons in Mouse Sires Promotes Paternal Behavior, Related to Figure 4 (A) Schematic illustration of the experimental design used in *in vivo* optogenetic experiments to stimulate MPOA *Gal*^+^ cells by photostimulation after expression of channelrhodopsin (ChR2). (B) Confocal Z stack image of a bilateral fiber-implanted *Gal*-Cre mouse sire, injected with DIO-eYFP. ft, optogenetic fiber track; oc, optic chiasm; 3V, third ventricle. (C) Confocal Z stack image of a bilateral fiber-implanted *Gal*-Cre mouse sire, injected with DIO-ChR2. (D) Illustration of the pup retrieval test design used in this experiment, typically including eight pieces of nesting material and six pups laid out in an open field arena. (E) Representative images at the beginning (t = 0 min) and end (t = 30 min) of a photostimulation trial in the pup retrieval test of a *Gal*-Cre mouse sire injected with DIO-ChR2 in MPOA. Photostimulation protocol was 10 Hz frequency, 5 msec pulse width, applied throughout the total recording period (30 min). (F) Quantification of pups retrieved (n = 18 trials per eYFP group, triplicates with 6 DIO-eYFP injected *Gal*-Cre mouse sires, two-tailed paired t test, p = 0.7462 between no photostimulation and photostimulation, n = 15 trials per ChR2 group, triplicates with 5 DIO-ChR2 injected *Gal*-Cre mouse sires, one-way repeated-measures ANOVA with Dunn’s post hoc test, p = 0.0028 between no photostimulation and photostimulation). (G) Quantification of latency to first pup retrieval (n = 18 trials per eYFP group, triplicates with 6 DIO-eYFP injected *Gal*-Cre mouse sires, one-way repeated-measures ANOVA with Dunn’s post hoc test, p = 0.5130 between no photostimulation and photostimulation, n = 15 trials per ChR2 group, triplicates with 5 DIO-ChR2 injected *Gal*-Cre mouse sires, one-way repeated-measures ANOVA with Dunn’s post hoc test, p = 0.0005 between no photostimulation and photostimulation). (H) Quantification of nest quality (n = 18 trials per eYFP group, triplicates with 6 DIO-eYFP injected *Gal*-Cre mouse sires, one-way repeated-measures ANOVA with Dunn’s post hoc test, p = 0.1216 between no photostimulation and photostimulation, n = 15 trials per ChR2 group, triplicates with 5 DIO-ChR2 injected *Gal*-Cre mouse sires, one-way repeated-measures ANOVA with Dunn’s post hoc test, p = 0.2981 between no photostimulation and photostimulation). ns; not significant, ^∗∗^p < 0.01, ^∗∗∗^p < 0.001. In box-and-whisker plots, center lines indicate medians, box edges represent the interquartile range, and whiskers extend to the minimal and maximal values. Duplicates or triplicates were collected and are presented as single dots from each animal. Each dot represents a readout from a single session in the pup retrieval test. All experiments presented in Figure S1 were performed in male mice.

Recent work has revealed that activation of MPOA neurons that express the neuropeptide galanin (*Gal*^+^) can drive parental behavior (Wu et al., 2014; Kohl et al., 2018), a finding that was confirmed in the male mouse in the present study (Figures S1A–S1H). To determine the effect of Prl on this parentally implicated MPOA subpopulation, we performed whole-cell patch-clamp recordings using *Gal*-tdTomato adult male virgin mice (Figure 4F). Application of Prl evoked a reversible depolarization in 11 of 13 recorded MPOA *Gal*^+^ neurons, paralleled by induction of action potential firing (Figure 4G). Increased intrinsic excitability was evident in the presence of Prl as augmented firing frequency in response to depolarization (Figure 4H) as well as an increase in the input resistance of these cells (Figure 4I). Parallel to these excitatory actions, application of Prl resulted in presynaptic changes with an increase in spontaneous excitatory input (Figure 4J) and a decrease in spontaneous inhibitory input (Figure 4K), effects reconcilable with overall stimulation of MPOA *Gal*^+^ cells. Investigation of the Prl-induced current in MPOA *Gal*^+^ neurons by voltage-clamp ramps combined with pharmacological manipulation (Figures 4L and 4M) identified K^+^ channels as mediating Prl’s postsynaptic depolarizing effect (Figure 4L, cesium + Prl). This effect appeared to be specifically mediated via closure of apamin-sensitive, Ca^2+^-dependent K^+^ channels of the small conductance (“SK”) type but not charybdotoxin-sensitive channels; i.e., of the large conductance (“BK”) type subclass (Figures 4L and 4M).

### Nonpaternal Rat and Paternal Mouse Sires

**Figure 5:**
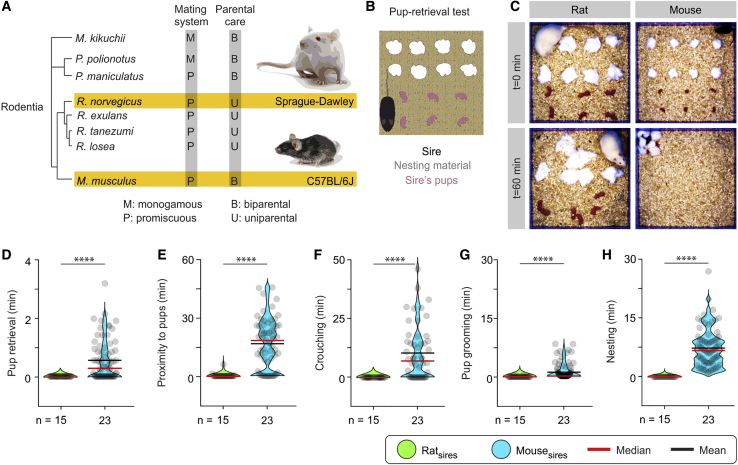
Sprague-Dawley Rat Sires Are Not Paternal, whereas C57BL/6 Mouse Sires Exhibit High Levels of Parental Behavior (A) Species of the order Rodentia employ several different combinations of strategies for mating and parental care. (B) The pup retrieval test was used to quantify parental care in rat and mouse sires. Typically, eight pieces of nesting material and six pups were laid out in an open field arena prior to introduction of the sire. (C) Representative images at the beginning (t = 0 min), and end (t = 60 min) of the pup retrieval test of a rat (left) and mouse (right) sire. (D–H) Quantification of indices of paternal investment in rat and mouse sires (D, pup retrieval duration; E, time spent in proximity to pups; F, time spent crouching over pups; G, time spent grooming pups; H, time spent nesting); n = 33 rat sire trials and 72 mouse trials from 15 rat and 23 mouse sires. Statistics for the rat versus mouse comparison are presented in all graphs. One-way repeated-measures ANOVA with Dunn’s post hoc test was used for all comparisons, rat versus mouse sires, p < 0.0001. Violin plots indicate the non-normal distribution of behaviors. Each dot represents a readout from a single session in the pup retrieval test. Duplicates or triplicates were collected and are presented as single dots for each animal. Note the higher performance score on all parameters for mice compared with rats. All experiments were performed in male rodents. ^∗∗∗∗^p < 0.0001. See also Figure S2.

Prl-mediated MPOA *Gal*^+^ cell activation coupled with the previously identified role of this area in parental behavior led us to test whether a difference occurs at the behavioral level in paternal care between the two species. Rats and mice have been described as uni- versus biparental (Egid and Lenington, 1983; Lee and Brown, 2002; Price and Belanger, 1977), respectively (Figure 5A), but strain differences and divergent evolution present in laboratory and wild strains have often led to discrepant observations (Samuels and Bridges, 1981; Jakubowski and Terkel, 1985; Leboucher, 1986). This made it necessary to assess parental care in the two species side by side.

**Figure S2:**
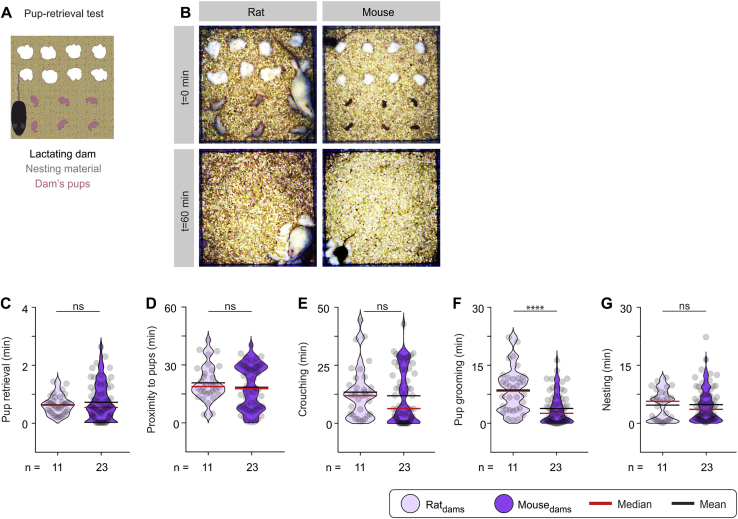
Sprague-Dawley Rat and C57BL/6 Mouse Lactating Dams Exhibit Similar Levels of Maternal Care, Related to Figure 5 (A) The pup retrieval test was used for quantification of parental care investment in rat and mouse lactating dams. Typically, eight pieces of nesting material and six pups were laid out in an open field arena, prior to introduction of the dam. (B) Representative images at the beginning (t = 0 min), and end (t = 60 min), in the pup retrieval test of a rat (left) and mouse (right) lactating dam. (C-G) Quantification of parameters relevant to paternal investment in rat and mouse lactating dams (n = 33 rat and 65 mouse lactating dam trials, from 11 rat and 23 mouse lactating dams). One-way repeated-measures ANOVA with Dunn’s post hoc test was used for all comparisons. (C) Pup retrieval duration (rat versus mouse dams, p = 0.8326). (D) Time spent in proximity to pups (rat versus mouse dams, p = 0.4416). (E) Time spent crouching over pups (rat versus mouse dams, p = 0.3018). (F) Pup grooming duration (rat versus mouse dams, p < 0.0001). (G) Time spent in nesting (rat versus mouse dams, p = 0.9880). ns; not significant, ^∗∗∗∗^p < 0.0001. Violin plots indicative of the non-normal distribution of behaviors. Each dot represents a readout from a single session in the pup retrieval test. Duplicates or triplicates were collected and are presented as single dots from each animal. All experiments presented in Figure S2 were performed in female rodents.

To compare parental care in male rats and mice, we used the pup retrieval test (Terkel et al., 1979; Wang and Storm, 2011), which was composed of a square arena containing shredded nesting material in one half and typically six pups in the other half (Figure 5B). Parental behavior was scored in repeated 1-h sessions for experienced sires and dams of the two species. Rat sires exhibited pup avoidance, in striking contrast to mouse sires, which performed parental behaviors, including nesting, pup retrieval, crouching, and pup grooming (Figures 5C–5H). Maternal behavior exhibited by the dams of the two species was similar in the majority of measurements (duration of pup retrieval, duration of proximity to pups, and crouching and nesting durations), with the exception of pup grooming, where rat lactating dams spent more time in comparison with mouse lactating dams (Figure S2).

The accumulated datasets to this point, although correlational, support the hypothesis that the TIDA neuron activity in males sets serum Prl levels, with consequent downstream activation of Prl-responsive brain nodes involved in parental behavior, such as the MPOA. These combined effects might influence or determine the expression of paternal care in a species. The next set of experiments was designed to causally assess the validity of this hypothesis.

### Administration of Prl Elicits Paternal Behavior in a Non-paternal Species

**Figure 6:**
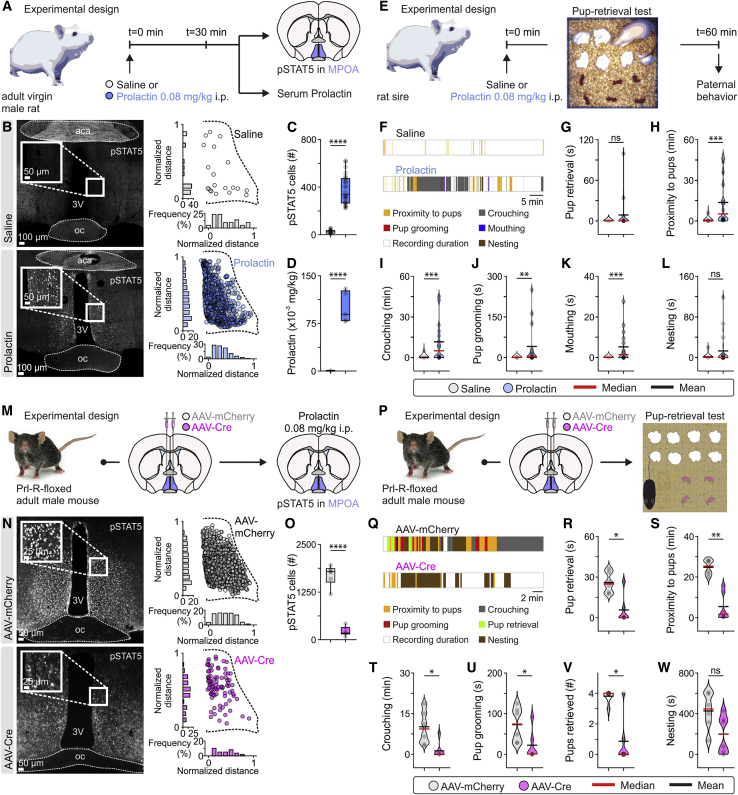
Following Sexual Experience, Prl Is Sufficient and Necessary for Paternal Behavior (A) Schematic of the experimental design used to determine whether systemic injection of Prl raises serum Prl levels and alters pSTAT5 immunofluorescence in the MPOA. (B) Representative confocal images and quantification of pSTAT5 immunofluorescence in the MPOA in saline-injected (top) and Prl-injected (bottom) adult virgin male rats. aca, anterior commissure. (C) Quantification of pSTAT5 cells in the MPOA in control and Prl-injected rats (n = 24 MPOA sections per group from 5 rats per group, two-tailed unpaired t test, p < 0.0001 between saline and Prl). (D) Quantification of serum Prl in control and Prl-injected rats (n = 5 per group, two-tailed unpaired t test, p < 0.0001 between saline and Prl). (E) Schematic of the experimental design used to assess whether i.p. administration of Prl can induce paternal behavior in sexually experienced rat sires. (F) Representative behavior raster plots of a rat sire in the pup retrieval test after saline (top) and Prl (bottom) injection. (G–L) Quantification of indices of paternal investment in rat sires (n = 13 and 16 trials with saline and Prl i.p. injection, respectively, one-way repeated-measures ANOVA with Dunn’s post hoc test). Note the improved performance on most parameters in response to Prl administration. (G) Pup retrieval duration; p = 0.0774 between saline and Prl. (H) Time spent in proximity to pups; p = 0.0001 between saline and Prl. (I) Crouching duration; p = 0.0003 between saline and Prl. (J) Pup grooming duration; p = 0.0012 between saline and Prl. (K) Mouthing duration; p = 0.0007 between saline and Prl. (L) Nesting duration; p = 0.0923 between saline and Prl. (M) Schematic of the experimental design used to determine the effect of conditional knockout of the Prl-R on pSTAT5 immunoreactivity in the MPOA. (N) Representative bright-field images and quantification of pSTAT5 immunoreactivity in the MPOA in AAV-mCherry-injected (control) and AAV-Cre-injected (Prl-R knockout) adult male mice. (O) Quantification of pSTAT5 cells in the MPOA in AAV-mCherry- and AAV-Cre-injected mice (n = 6 bilateral MPOA sections per group from 6 mice per group, two-tailed unpaired t test, p < 0.0001 between AAV-mCherry and AAV-Cre). (P) Schematic of the experimental design used to assess whether MPOA Prl-R knockout in mouse sires influences paternal behavior. (Q) Representative behavior raster plots of two individual mouse sires in the pup retrieval test after AAV-mCherry (top) and AAV-Cre (bottom) injection, respectively, in the MPOA. (R–W) Quantification of indices of paternal investment in mouse sires (n = 6 trials/mice/group with AAV-mCherry and AAV-Cre MPOA injection, respectively; two-sided Mann-Whitney *U* test was used for all comparisons). (R) Duration of pup retrieval behavior exhibited by mouse sires (AAV-mCherry versus AAV-Cre, p = 0.0195). (S) Time spent in proximity to pups (AAV-mCherry versus AAV-Cre, p = 0.0022). (T) Duration of crouching above pups (AAV-mCherry versus AAV-Cre, p = 0.0130). (U) Duration of pup grooming behavior exhibited by mouse sires toward pups (AAV-mCherry versus AAV-Cre, p = 0.0346). (V) Total number of pups retrieved at the end of the test (maximum number of pups = 4, AAV-mCherry versus AAV-Cre, p = 0.0152). (W) Duration of nesting behavior exhibited by mouse sires (AAV-mCherry versus AAV-Cre, p = 0.0931). ^∗^p < 0.05, ^∗∗^p < 0.01, ^∗∗∗^p < 0.001, ^∗∗∗∗^p < 0.0001. Each dot represents a readout from a single session in the pup retrieval test. Duplicates or triplicates were collected and are presented as single dots for each animal. Violin plots indicate the non-normal distribution of behaviors. All experiments were performed in male rodents. See also Figures S3, S4, and S5.

**Figure S3:**
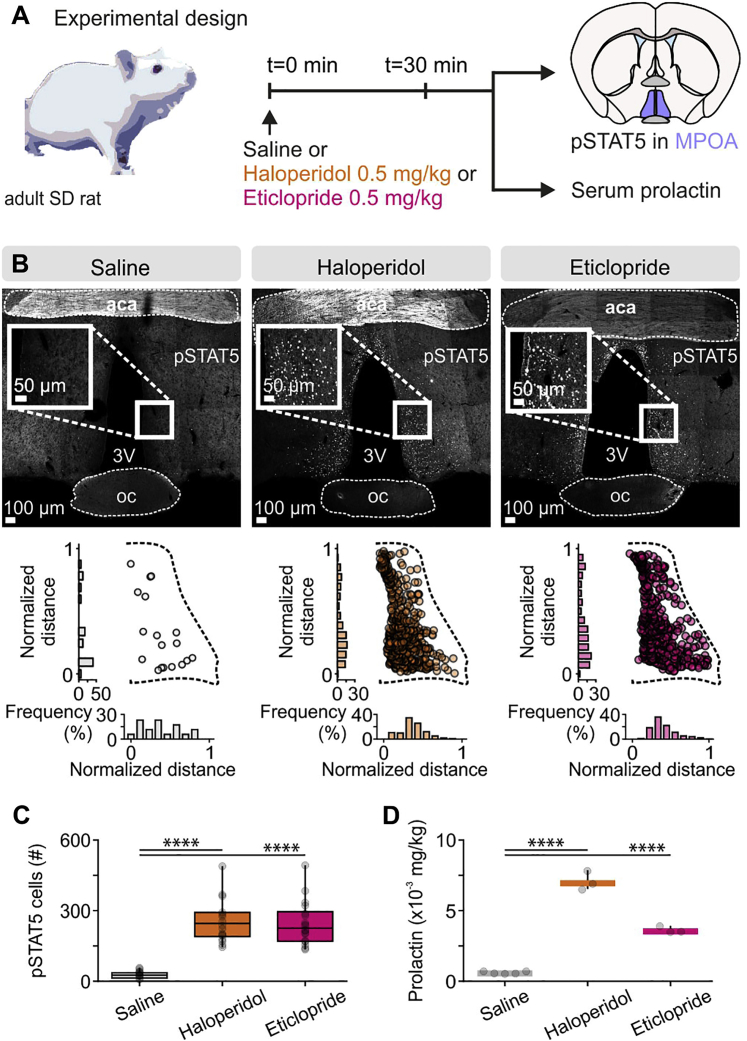
Injection of Dopamine D2 Receptor Antagonists Results in Increased Serum Prl Levels and MPOA Prl-R Activation in Male Rats, Related to Figure 6 (A) Experimental design used to assess whether i.p. injection of the dopamine D2-type receptor antagonists haloperidol and eticlopride, raises serum prolactin levels and increases pSTAT5 immunofluorescence in the MPOA. (B) Representative confocal images and quantification of pSTAT5 immunofluorescence in the MPOA, in saline, haloperidol and eticlopride injected adult virgin male rats. aca, anterior commissure; oc, optic chiasm; 3V, third ventricle. (C) Quantification of pSTAT5 cells in MPOA (n = 24 MPOA sections from 5 rats for the saline group and n = 18 sections from 3 rats for the haloperidol and eticlopride groups, two-tailed unpaired t test, p < 0.0001 between saline and haloperidol; p < 0.0001 between saline and eticlopride). (D) Quantification of serum prolactin (n = 5 for saline, n = 3 for haloperidol and n = 3 for the eticlopride group, two-tailed unpaired t test, p < 0.0001 between saline and haloperidol; p < 0.0001 between saline and eticlopride). ^∗∗∗∗^p < 0.0001. In box-and-whisker plots, center lines indicate medians, box edges represent the interquartile range, and whiskers extend to the minimal and maximal values. In bar graphs, data are expressed as mean ± s.e.m. All experiments presented in Figure S3 were performed in male rats.

First, we addressed whether parental care behavior could be triggered in the normally non-parental male rat by artificially elevating circulating Prl. This necessitated initially determining whether the male rat MPOA is sensitive to Prl, because the naturally occurring low serum Prl levels (Figure 3D) could be paralleled by an absence of Prl-Rs. To test whether circulating Prl can induce Prl-R activation in the rat MPOA, we administered 0.08 mg/kg Prl or saline (vehicle) intraperitoneally (i.p.) and collected tail blood 30 min post-administration to determine serum Prl and brain tissue to assess pSTAT5 immunofluorescence (Figure 6A). Administration of Prl raised serum Prl levels from 0.61 ± 0.04 × 10^−3^ mg/kg to 100.60 ± 10.96 × 10^−3^ mg/kg (Figure 6D), a serum concentration typically found in rat and mouse lactating dams (Voloschin et al., 1998; Guillou et al., 2015), and induced an increase in pSTAT5 immunofluorescent cells from 26.63 ± 2.70 to 381.90 ± 25.38 MPOA cells per section (Figures 6B and 6C). Last, to trigger endogenously sourced hypersecretion of Prl, virgin male rats received an i.p. injection of the dopamine D2 receptor-blocking antipsychotic haloperidol (Banasikowski and Beninger, 2012; Hillegaart and Ahlenius, 1987) or eticlopride (Svenningsson et al., 2000; Tang and Dani, 2009), a selective D2-type receptor antagonist, known to induce hyperprolactinemia in research models and in the clinic (Spitzer et al., 1998; Arita and Kimura, 1986; Morgan et al., 1984). These manipulations increased both serum Prl levels and the number of pSTAT5-positive cells in the rat MPOA (Figure S3), indicative of a functional lactotropic axis in the male rat. These findings provide evidence that elevated circulating Prl can reach the MPOA in a manner consistent with direct mediation via the Prl-R, in agreement with demonstrations that at least some of the MPOA galanin neurons lie outside of the blood-brain barrier (Rajendren et al., 2000).

**Figure S4:**
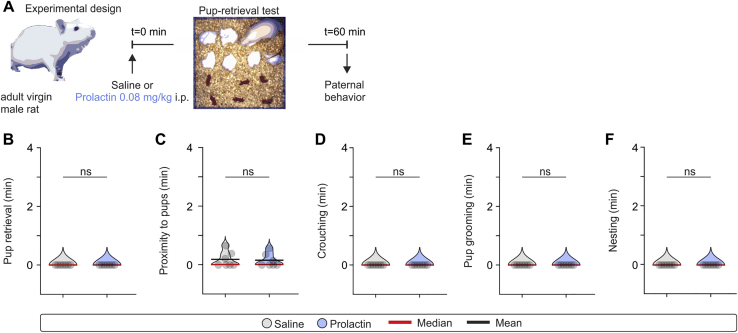
In the Absence of Sexual Experience, Prl Administration Does Not Induce Paternal Behavior in the Rat, Related to Figure 6 (A) Schematic of the experimental design used to assess whether i.p. administration of prolactin can induce paternal behavior in the virgin male rat. (B-F) Quantification of indices of parental behavior in virgin male rats following administration of i.p. injection of saline or prolactin (n = 7 trials per group, from 7 rats). Two-sided Mann–Whitney U test was used for comparisons between saline and prolactin. (B) Pup retrieval duration (saline versus prolactin, p > 0.9999). (C) Time spent in proximity to pups (saline versus prolactin, p = 0.8776). (D) Time spent crouching over pups (saline versus prolactin, p > 0.9999). (E) Pup grooming duration (saline versus prolactin, p > 0.9999). (F) Time spent in nesting (saline versus prolactin, p > 0.9999). ns; not significant. Violin plots indicative of the non-normal distribution of behaviors. Each dot represents a readout from a single session in the pup retrieval test. All experiments presented in Figure S4 were performed in male rats.

The above observations raised the possibility that increasing serum Prl, through consequent activation of MPOA neurons, can induce paternal behavior in rat sires. To test this hypothesis, we performed repeated pup retrieval tests in previously identified non-paternal experienced rat sires following saline and Prl administration (Figure 6E). Similar to what was observed in baseline behavioral recordings (Figure 5), saline-injected rat sires exhibited pup avoidance (Figures 6F–6L; Video S1). In striking contrast, Prl-injected rat sires exhibited greater amounts of passive aspects of paternal care, such as crouching and increased time in proximity to the pups, and, importantly, active paternal behaviors, such as pup grooming and mouthing (Figures 6F–6L; Video S2). Another salient observation was that Prl administration did not induce paternal behavior in virgin male rats (Figure S4), indicating that Prl’s effect on paternal behavior is conditional on having prior sexual experience. Together, these data suggest that Prl acts as a permissive gain control signal in the expression of paternal care, with increased Prl levels able to acutely evoke sequences of paternal behavior in fathers of a non-paternal species.

Video S1. Absence of Paternal Care in Rat Sires following Acute Administration of Saline, Related to Figure 6

Video S2. Induction of Paternal Care in Rat Sires following Acute Administration of Prl at 0.08 mg/kg, Related to Figure 6

### Acute Conditional Deletion of Prl-R in the Mouse MPOA Disrupts Paternal Behavior

Although paternal behavior can be evoked by Prl administration in rat sires (Figures 6E–6L), it remains unknown whether Prl is necessary for sexually experienced males to exhibit parental behavior. Furthermore, the brain area(s) critical to Prl’s ability to drive paternal behavior remain unidentified. The MPOA is a prime candidate for testing the role of Prl in paternal behavior because its influence on parental behavior is well established (Kuroda and Numan, 2014; Numan et al., 1977; Wu et al., 2014), it exhibits prominent expression of the Prl-R (Brown et al., 2010, 2016), and, as demonstrated above, MPOA *Gal*^*+*^ neurons are directly excited by Prl.

**Figure S5:**
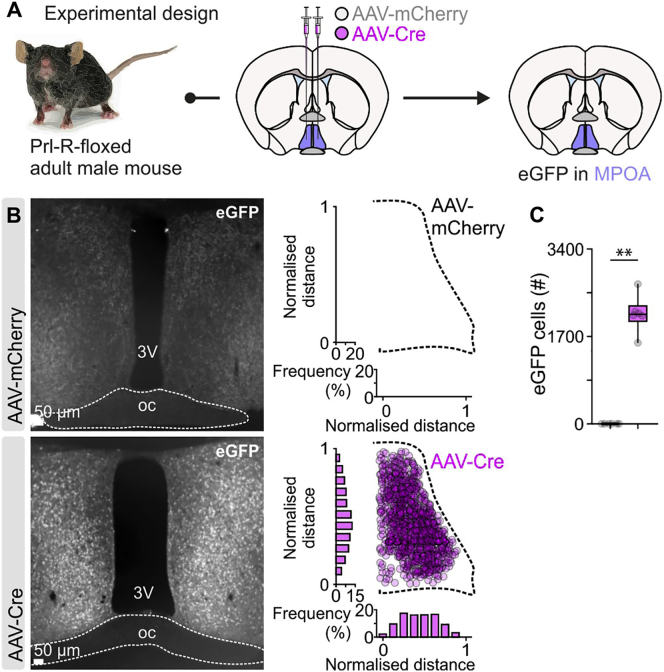
Validation of Prl-R Gene Deletion, Related to Figure 6 (A) Schematic of the experimental design used to determine if stereotactic injection of AAV-Cre in the MPOA of prolactin-receptor floxed mice leads to deletion of the prolactin receptor gene. As Cre-mediated deletion of the prolactin gene involves simultaneous inversion of the prolactin receptor gene and eGFP expression, eGFP fluorescence can serve as a marker of the injection site and successful recombination. (B) Representative brightfield images and quantification of eGFP immunoreactivity in the MPOA, in AAV-mCherry (control) and AAV-Cre (prolactin receptor knockout) injected adult male mice. oc, optic chiasm; 3V, third ventricle. Note the absence of eGFP immunoreactivity in the control (AAV-mCherry injected) mice, in contrast to the expression of eGFP (a marker of the prolactin receptor gene deletion) in the MPOA of AAV-Cre injected mice. (C) Quantification of eGFP cells in the MPOA in AAV-mCherry and AAV-Cre-injected mice (n = 6 bilateral MPOA sections per group from 6 mice per group, two-sided Mann–Whitney U test, p = 0.0022 between AAV-mCherry and AAV-Cre). ^∗∗^p < 0.01. In box-and-whisker plots, center lines indicate medians, box edges represent the interquartile range, and whiskers extend to the minimal and maximal values. All experiments presented in Figure S5 were performed in male mice.

To test this hypothesis, we performed Cre-mediated conditional deletion of the Prl-R in the MPOA, using targeted bilateral injections of adeno-associated virus (AAV)-Cre into Prl-R floxed animals (Brown et al., 2017). Successful recombination of the Prl-R resulted in a strong immunoreactive signal for EGFP, whereas in control mice (injected with AAV-mCherry), EGFP immunoreactivity was absent, indicating no recombination of the Prl-R gene (Figure S5). To further confirm the absence of Prl-R activity in AAV-Cre-injected mice, pSTAT5 activity was assessed following an acute dose of exogenous Prl by i.p. administration. Although control mice showed high levels of pSTAT5 immunoreactivity in the MPOA, mice with an MPOA-specific deletion of Prl-R had dramatically reduced numbers of pSTAT5-immunoreactive cells following Prl administration (Figures 6M–6O).

To test the effect of MPOA-restricted Prl-R deletion on paternal behavior, Prl-R floxed adult male mice were injected with AAV-mCherry or AAV-Cre in the MPOA. Following successful mating with a female, paternal behavior was tested on pups on post-natal day 3. AAV-Cre treated animals demonstrated major impairments in all aspects of paternal behavior tested, with the exception of nesting (Figures 6P–6W). The possibility of non-specific effects of the Cre construct used here was assessed in a previous study of maternal behavior (Brown et al., 2017). These experiments showed that no reduction of Prl-R activity or parental behavior occurred when the same AAV-Cre was injected at the same dose and the same stereotactic coordinates in wild-type animals, albeit in female and not male mice. Thus, the results shown above are most likely caused by conditional and specific deletion of the Prl-R rather than adverse effects of the Cre construct itself. These results support the hypothesis that Prl-responsive MPOA neuron activity is critical for the expression of paternal care.

### Optogenetic Control of Hypothalamic Oscillations and Paternal Behavior

While the above findings suggest that Prl has a permissive (i.e., following mating) but necessary role in paternal behavior, a causal link between the pattern of activity of TIDA neurons, - the “control center” for Prl - and serum Prl levels and paternal behavior remained to be established.

**Figure 7:**
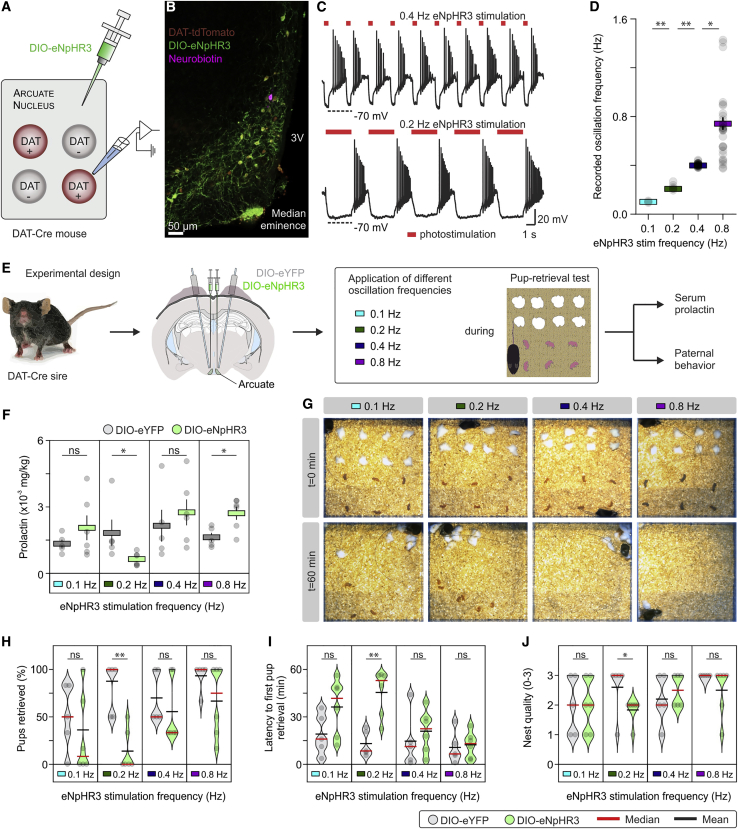
Optogenetic Tuning of TIDA Oscillation Frequency Scales Serum Prl and Paternal Behavior (A) Schematic illustration of Cre-dependent introduction of eNpHR3 in DAT^+^ cells in the dmArc to control the electrical activity of TIDA neurons. (B) Confocal z stack image of a section from the arcuate nucleus of an eNpHR3-injected DAT-tdTomato mouse, showing a recorded and neurobiotin-filled (purple) TIDA neuron. (C) *In vitro* whole-cell recording showing how photoinhibition at regular intervals results in robust oscillation frequency control in mouse TIDA neurons. (D) Group data from recordings as illustrated in (C); photoinhibition-imposed frequency versus recorded oscillation frequency (n = 30 trials from 10 cells from 6 animals, comparisons were performed with Kruskal-Wallis test followed by Dunn’s test; p = 0.0051 between 0.1 Hz and 0.2 Hz; p = 0.0019 between 0.2 Hz and 0.4 Hz, p = 0.0306 between 0.4 Hz and 0.8 Hz). (E) Schematic of the experimental design used for optogenetic control of TIDA activity during the pup retrieval test, followed by tail blood sample collection and quantification of serum Prl. (F) Serum Prl 1 h after initiation of *in vivo* photoinhibition during the pup retrieval test in eYFP-injected (n = 5) and eNpHR3-injected (n = 6) mouse sires. Prl levels drop significantly when TIDA neurons are driven at the slow, rat-like frequency (and rise with the supra-endogenous frequency) (two-tailed unpaired t test; p = 0.2899 between eYFP and eNpHR3 at 0.1 Hz, p = 0.0316 between eYFP and eNpHR3 at 0.2 Hz, p = 0.5253 between eYFP and eNpHR3 at 0.4 Hz, and p = 0.0170 between eYFP and eNpHR3 at 0.8 Hz). (G) Representative images at the beginning (t = 0 min) and end (t = 60 min) of the pup retrieval test of mouse sires exposed to different photoinhibition frequencies. Note impaired retrieval when the slow (0.2 Hz) frequency is applied. (H–J) Quantification of indices of paternal investment (n = 5–6 trials/mice/group with eYFP or eNpHR3 TIDA neuron transduction, respectively; two-sided Mann-Whitney *U* test was used for all comparisons). Each dot indicates an individual animal in trials with or without photoinhibition. (H) Successful pup retrieval. (I) Latency to first pup retrieval. (J) Nest quality. ^∗^p < 0.05, ^∗∗^p < 0.01. In bar graphs, data are expressed as mean ± SEM. In box-and-whisker plots, center lines indicate medians, box edges represent the interquartile range, and whiskers extend to the minimal and maximal values. In violin plots, center lines indicate medians, and dashed lines represent interquartile range. All experiments were performed in male mice. See also Figures S6 and S7.

To test the hypothesis that defined TIDA neuron rhythms determine serum Prl and, as a consequence, paternal behavior, we optogenetically recreated the oscillation frequencies recorded in the TIDA system during the pup retrieval test in mouse sires. Distinct frequencies were applied via photostimulation (Figures 7A–7D) in repeated trials of the pup retrieval test, during which paternal behavior was recorded, and, at the end of the test, serum Prl was sampled (Figure 7E). For this experiment, we used optogenetic inhibition (using eNpHR3 constructs) rather than stimulation (using ChR2) because TIDA neurons are spontaneously active and therefore best entrained by periodic hyperpolarization, whereas depolarization risks interfering with ongoing discharge. This strategy yielded high-fidelity control of TIDA oscillation frequency (Figure 7C).

**Figure S6:**
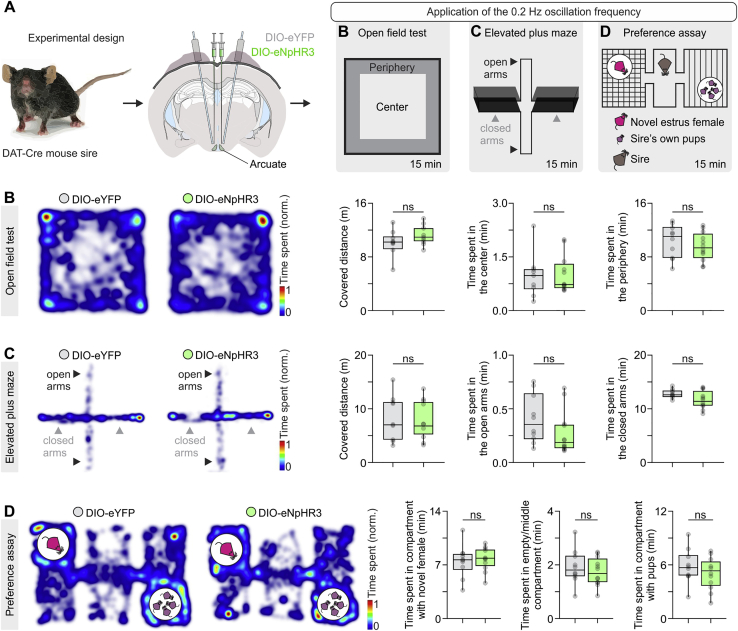
The Optogenetic Regimen that Impairs Expression of Paternal Behavior in Mouse Sires Does Not Lead to Changes in Locomotion, Anxiety, or Pup Preference, Related to Figure 7 (A) Schematic of the experimental design used for the optogenetic control of TIDA activity to impose the 0.2 Hz frequency, during behavioral investigations of locomotion, anxiety and pup preference. (B) Use of the open field test and quantification of locomotion, thigmotaxis (time spent in the periphery) and time spent in the center of the arena. Distance covered (n = 10 trials from 5 eYFP mice versus n = 12 trials from 6 eNpHR3 mice, two-tailed unpaired t test, p = 0.1121). Time spent in the center (n = 10 trials from 5 eYFP mice versus n = 12 trials from 6 eNpHR3 mice, two-sided Mann–Whitney U test, p = 0.9742). Time spent in the periphery (n = 10 trials from 5 eYFP mice versus n = 12 trials from 6 eNpHR3 mice, two-tailed unpaired t test, p = 0.4163). (C) Use of the elevated plus maze for the quantification of anxiety-display. Distance covered (n = 10 trials from 5 eYFP mice versus n = 12 trials from 6 eNpHR3 mice, two-tailed unpaired t test, p = 0.7970). Time spent in the open arms (n = 10 trials from 5 eYFP mice versus n = 12 trials from 6 eNpHR3 mice, two-sided Mann–Whitney U test, p = 0.0804). Time spent in the closed arms (n = 10 trials from 5 eYFP mice versus n = 12 trials from 6 eNpHR3 mice, two-tailed unpaired t test, p = 0.0538). (D) Use of a behavioral assay aimed to test the preference of the mouse sire to spend time with a novel estrus female or his own pups. Time spent in compartment with the novel estrus female (n = 10 trials from 5 eYFP mice versus n = 12 trials from 6 eNpHR3 mice, two-tailed unpaired t test, p = 0.6530). Time spent in the middle/empty compartment (n = 10 trials from 5 eYFP mice versus n = 12 trials from 6 eNpHR3 mice, two-tailed unpaired t test, p = 0.4712). Time spent in the compartment with pups (n = 10 trials from 5 eYFP mice versus n = 12 trials from 6 eNpHR3 mice, two-tailed unpaired t test, p = 0.3275). ns; not significant. In box-and-whisker plots, center lines indicate medians, box edges represent the interquartile range, and whiskers extend to the minimal and maximal values. All experiments presented in Figure S6 were performed in male mice.

Application of the slow 0.2-Hz frequency (found in the rat) resulted in a decrease in serum Prl to 0.6484 ± 0.2942 × 10^−3^ mg/kg in eNpHR3-expressing animals versus 1.828 ± 0.5991 × 10^−3^ mg/kg in eYFP-expressing (control) animals (Figure 7F). Importantly, photostimulation at 0.2 Hz impaired expression of paternal care, as quantified by the total number of pups retrieved, latency to first pup retrieval, and nest quality (Figures 7G–7J). However, optogenetic application of the 0.2-Hz frequency (i.e., the optogenetic regimen that impairs paternal behavior) did not alter locomotion, anxiety display, and preference and motivation parameters (Figure S6).

**Figure S7:**
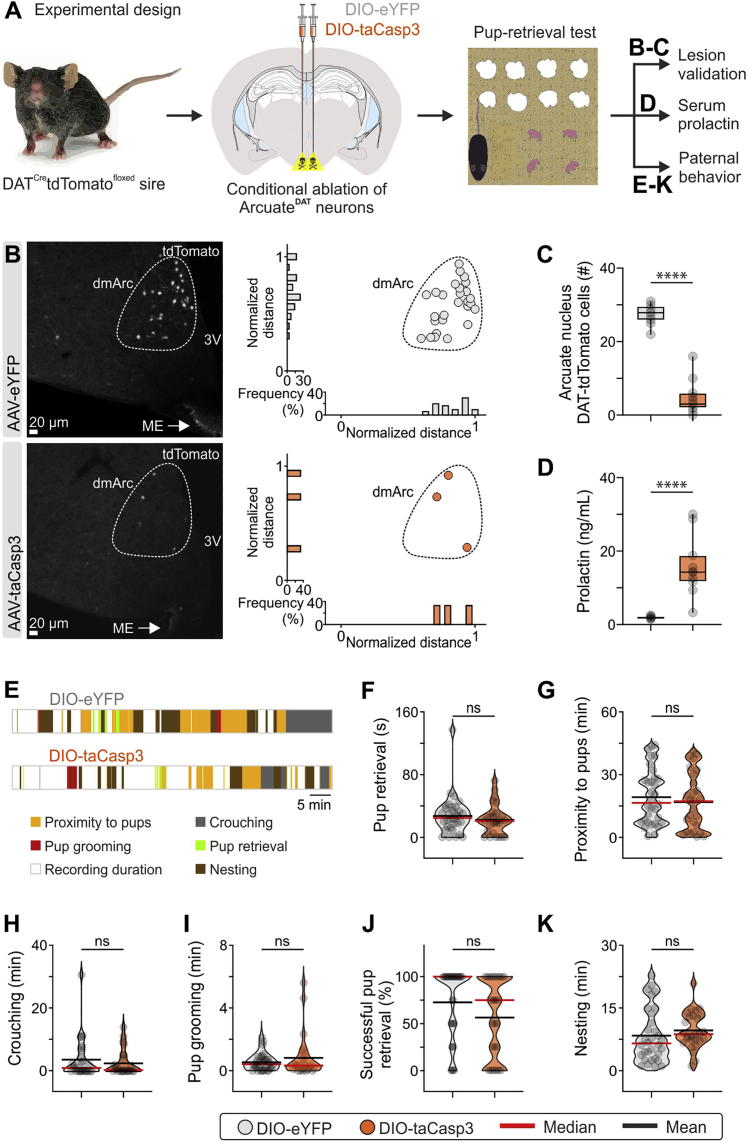
Conditional Ablation of TIDA Neurons in Mouse Sires Leads to Elevated Serum Prl but No Changes in Parental Behavior, Related to Figure 7 (A) Schematic of the experimental design used for the conditional ablation of TIDA neurons, and follow-up measurements. (B) Representative confocal images and quantification of DAT-tdTomato neuron number in the dorsomedial arcuate nucleus (dmArc), in AAV-DIO-eYFP (control) and AAV-DIO-taCasp3 (for TIDA neuron ablation) injected mouse sires. ME, median eminence; 3V, third ventricle. Note the near-complete absence of tdTomato immunofluorescence in the ablated arcuate nucleus (AAV-DIO-taCasp3 injected) mice. (C) Quantification of DAT-tdTomato cells in the arcuate in AAV-DIO-eYFP and AAV-DIO-taCasp3-injected mice (n = 11-12 bilateral arcuate nucleus sections per group from 11-12 mice per group, two-sided Mann–Whitney U test, p < 0.0001 between AAV-DIO-eYFP and AAV-DIO-taCasp3). (D) Quantification of serum prolactin in control and taCasp3 arcuate-injected mice (n = 11-12 mice per group, two-tailed unpaired t test, p < 0.0001 between AAV-DIO-eYFP and AAV-DIO-taCasp3). (E) Representative behavior raster plots of two individual mouse sires in the pup retrieval test, one-month post AAV-DIO-eYFP (top) and AAV-DIO-taCasp3 (bottom) arcuate nucleus transduction. (F-K) Quantification of indices of paternal investment in mouse sires (n = 2-3 trials/mouse/group with AAV-DIO-eYFP and AAV-DIO-taCasp3 arcuate nucleus injection, respectively. Two-sided Mann–Whitney U test was used for all comparisons, apart from Pup retrieval duration, Nesting and Proximity to pups in which two-tailed unpaired t test was used). (F) Duration of pup retrieval behavior exhibited by mouse sires (AAV-DIO-eYFP versus AAV-DIO-taCasp3, p = 0.3411). (G) Time spent in proximity to pups (AAV-DIO-eYFP versus AAV-DIO-taCasp3, p = 0.4953). (H) Duration of crouching above pups (AAV-DIO-eYFP versus AAV-DIO-taCasp3, p = 0.7585). (I) Duration of pup grooming behavior exhibited from mouse sires toward pups (AAV-DIO-eYFP versus AAV-DIO-taCasp3, p = 0.8765). (J) Total number of pups retrieved at the end of the test (maximum number of pups = 4, AAV-DIO-eYFP versus AAV-DIO-taCasp3, p = 0.1000). (K) Duration of nesting behavior exhibited by mouse sires (AAV-DIO-eYFP versus AAV-DIO-taCasp3, p = 0.3974). ns; not significant, ^∗∗∗∗^p < 0.0001. In box-and-whisker plots, center lines indicate medians, box edges represent the interquartile range, and whiskers extend to the minimal and maximal values. All experiments presented in Figure S7 were performed in male mice.

Application of the fast 0.4-Hz frequency (found in the mouse) had no effect on serum Prl, and mouse sires exhibited levels of paternal care similar to those recorded under baseline conditions (Figures 5C–5H and 7G–7J). Similarly, the 0.1-Hz and 0.8-Hz optogenetic regimens did not alter paternal behavior. Interestingly, a further increase in serum Prl in mouse sires via optogenetically applied fast oscillations on TIDA neurons (0.8 Hz; Figure 7F) or targeted Cre-dependent genetic ablation of TIDA cells (Figure S7) did not lead to further enhancement of paternal behavior.

These data provide evidence that defined TIDA neuron oscillation frequencies directly tune Prl release and, farther downstream, paternal behavior.

## Discussion

Nursing and protection of the vulnerable newborn is crucial for the survival of offspring but also to ensure the perpetuation of a species. Across species, animals practice specific social behaviors to optimize the well-being and survival of their young (Dulac et al., 2014; Zilkha et al., 2017). A central aspect of parental behavior that exhibits substantial cross-species variability is the extent of paternal involvement. Paternal behavior can also differ considerably between related species, such as the mouse and the rat, as shown here in a direct comparison (present data), in agreement with earlier descriptions of non-parental male rats (Lonstein and De Vries, 2000) and parental male laboratory mice (Svare et al., 1977; Priestnall and Young, 1978; Mak and Weiss, 2010; Scott et al., 2015). Although select brain regions (in particular the MPOA), neuromodulators, and hormones have been associated with expression of parenting in the male, current knowledge has yet failed to provide an integrated systems-level, circuit-based understanding of paternal behaviors (Kohl and Dulac, 2018).

Here we show that a recently discovered species difference in TIDA neuron oscillations (Stagkourakis et al., 2018) translates into distinct release volumes of dopamine toward the pituitary. This difference is correlated with high serum Prl and MPOA activation in the male mouse in contrast to the male rat: a hormonal and brain activation state that leads to opposing approaches to paternal care. Optogenetic control of TIDA oscillations suggests that frequency tuning of this network rhythm causally determines circulating Prl levels and parental behavior in the male. Furthermore, by exogenously raising serum Prl levels in rats, paternal behaviors can be induced in non-parental sires (but not in virgin males). Conditional knockout of the Prl-R in the MPOA confirmed that Prl action here is required for normal expression of paternal behavior in male mice. These data reveal a frequency-tuned brain-endocrine-brain circuit that can serve as a gain control mechanism for paternal care of offspring.

### Frequency Coding of Prl Release

Although neuronal oscillations are found in a multitude of systems across the neuraxis and are now broadly viewed as a fundamental mode of operation for neuronal computations (Lisman and Buzsáki, 2008; Singer and Gray, 1995), it remains poorly understood how different frequencies affect the output of a circuit. We recently demonstrated that the slow and fast oscillation frequencies of rat and mouse TIDA neurons, respectively, can be explained by dense and strong gap junction coupling in the former and complete absence of electrical synapses in the latter (Stagkourakis et al., 2018). In the current study, oscillation frequencies spanning a narrow interval (that includes the rat and mouse rhythms) were found to yield markedly different dopamine release. The failure to sustain high release at 0.4 Hz or higher is likely most parsimoniously explained as an inability to match exocytosis (demand) with reuptake and/or *de novo* synthesis (supply) of dopamine. The issue of how network oscillation frequency, although relevant to many brain systems, shapes the output of an ensemble is largely unexplored. However, a partly related issue regarding stimulus-secretion coupling in different discharge patterns (i.e., single spikes versus bursting) has been explored in midbrain dopamine and neuroendocrine magnocellular systems, where vesicle release is more efficient in bursting than in tonic discharge mode (Dutton and Dyball, 1979; Gonon, 1988).

### Species-Specific Prl Concentrations Determine Paternal Behavior in Offspring

We found that circulating Prl is severalfold higher in male mice than in rats. Prl measurements, using different techniques, are common in the literature (Aquino et al., 2017; Guillou et al., 2015), but, to our knowledge, the two species have not been measured in parallel in one study. Intriguingly, despite the clear differences in Prl levels in male rats and mice, neither species showed a difference between virgins and sires. These results are in contrast to some distinctly biparental species where Prl levels are elevated in fathers; e.g., *Peromyscus* (Gubernick and Nelson, 1989) and marmosets (Dixson and George, 1982). It cannot be excluded that laboratory mice, which have been bred selectively through multiple generations to optimize procreant success (which may include not only aspects of fertility but also parenting behaviors; Wu et al., 2014) have evolved distinct features of the reproductive axis. Nevertheless, the insensitivity of Prl to paternal status in male C57BL/6 mice (present results), with their proficiency for paternal care (Mak and Weiss, 2010; Scott et al., 2015; Svare et al., 1977), which is also expressed in the presence of the mother of its offspring (Priestnall and Young, 1978), suggests that the role of the hormone is permissive (rather than sufficient in exclusivity). This conclusion is further supported by the presence of a strong pSTAT5 signal in virgin and sire male mice alike, as shown here. Notably, male adult responses to pups depend on experience in this species; prior to fathering, pup encounters commonly elicit infanticide in male mice, a behavior that abates after successful reproduction (vom Saal, 1985). It is expected that additional factors contribute to establishing paternal care.

However, a causal role of Prl in male parenting is highlighted by our demonstration that acute administration of the hormone is sufficient to induce several aspects of pup care in non-paternal rat sires (in agreement with results by Sakaguchi et al., 1996). These actions, taking place with short latency (within an hour) are unlikely to involve the neurogenesis-promoting properties of Prl (Hunt and Jacobson, 1971; Shingo et al., 2003), a phenomenon that has been implicated in the ability of male mice to recognize their pups (Mak and Weiss, 2010). The basal circulating Prl levels, sufficiently high for stimulating parental care in mouse sires, are indicative of a preadaptation to fatherhood, distinct from the dramatic surge of Prl that occurs in late pregnancy in female mice, persisting into the nursing period (Guillou et al., 2015), similar to human mothers (Frantz, 1973b). A “mating-insensitive” Prl tone in the male does not exclude the possibility that scaling of oscillation frequency can yield individual differences in Prl (and, by inference, parental behavior) or that dynamic tuning of frequency within an individual can occur to modulate behavioral expression. Our demonstration that optogenetic manipulation of TIDA oscillations can lower and elevate serum Prl suggests that TIDA rhythms can act as a gain control for Prl in the male. However, it is important to note the non-linearity in the mechanism of action of Prl because a further increase in serum Prl in mouse sires via optogenetically applied fast oscillations on TIDA neurons (Figure 7F) or via targeted Cre-dependent genetic ablation of TIDA cells (Figure S7) does not further enhance paternal behavior. Last, in female rodents, we have reported a switch in TIDA oscillation frequency with pregnancy and lactation (Briffaud et al., 2015; Thörn Pérez et al., 2020), indicating that the neuroendocrine dopamine network may be subject to frequency tuning as adaptive demands for Prl change.

### The TIDA-Prl Axis and the Final Common Pathway for Parental Behavior

The actions of Prl on paternal care are mediated through the MPOA; we show that (1) phosphorylation of STAT5, a sensitive indicator of the Prl-R pathway (Gouilleux et al., 1994), is greater in male mice compared with rats; (2) this activation increases when serum Prl is increased; (3) MPOA *Gal*^*+*^ neurons are directly (as well as indirectly) stimulated by Prl; and (4) Prl-R deletion specifically from MPOA neurons decreases paternal behavior. The MPOA, especially galanin neurons, has emerged as a final common pathway for parental behavior (Wu et al., 2014), and Prl-R neurons in the MPOA are necessary brain targets for Prl to drive maternal care (Brown et al., 2017). Indeed, a recent spatial transcriptomics study (Moffitt et al., 2018) demonstrated expression of the Prl-R in galanin neurons of the MPOA, but it should be noted that the receptor is also observed in non-galaninergic neurons of this nucleus, suggesting that additional MPOA populations may contribute to the parental effects of Prl. In turn, these neurons appear to recruit a broad panel of downstream CNS targets that generate specific components of parenting (Kohl et al., 2018). Here we show that Prl excites presynaptic inputs of MPOA *Gal*^+^ neurons (Figures 4J and 4K) and that it mediates its actions at least in part via a post-synaptic effect (Figures 4L and 4M). Identification of SK channel inhibition as the mechanism underlying the post-synaptic action of Prl (Figures 4L and 4M) can explain the emergence of high-frequency firing in MPOA *Gal*^+^ neurons in the presence of the hormone (Figure 4H; Ellis et al., 2007; Yen et al., 1999) and, consequently, increased network output. It remains to be determined whether Prl acts on all MPOA *Gal*^*+*^ subsets or whether it is biased toward some of the functional subgroups identified by Kohl et al. (2018). Among the neurons recorded in our study, Prl responsiveness was nearly uniform, but a correlation with downstream targets of these cells will be an intriguing subject for future studies. Our data also do not exclude additional brain targets for Prl in control of parental behavior. There are several populations expressing the Prl-R that form an interconnected network, each of which contributes to maternal care performance (Brown et al., 2017). The role of this network in the male is one of the questions prompted by the present results.

### Conclusion

In conclusion, the findings presented here reveal how distinct coupling schemes within a hypothalamic network, resulting in distinct membrane potential oscillation frequencies, can set the tone in a hormonal axis and, thus, determine species-specific parental strategies. By using two related model species characterized by bi- and uniparental strategies, respectively, we provide causal evidence that Prl can act as a driver of paternal behavior in rodents and that factors that influence the rhythm of TIDA neurons may serve as gain control checks for parental behavior in fathers. Although the electrical properties of the human TIDA system have remained inaccessible to experimental analysis, the similarities in the role of dopamine in Prl control in humans and rodents (Frantz, 1973a; Ben-Jonathan et al., 2008) suggest the possibility that similar mechanisms may be evolutionarily conserved across species.

## STAR★Methods

### Key Resources Table

**Table:** 

REAGENT or RESOURCE	SOURCE	IDENTIFIER
**Antibodies**		

Mouse monoclonal anti-TH	Millipore	Cat# MAB318
Rabbit polyclonal anti-TH	Millipore	Cat# AB152
Rabbit monoclonal anti-pSTAT5 (Tyr694)	Cell Signaling	Cat# 9359L; RRID: C11C5
Rabbit monoclonal anti-NeuN	Cell Signaling	Cat# 24307S; RRID: D4G40
Rabbit monoclonal anti-DsRed	Takara	Cat# 632392
Anti-GFP rabbit serum	Invitrogen	Cat# A-6455; RRID: AB_221570
Chicken polyclonal anti-GFP	Aves Labs, Inc.	Cat# GFP-1010; RRID: AB_2307313
Donkey anti-Mouse IgG- Alexa Fluor 488	ThermoFisher	Cat# A-21202; RRID: AB_141607
Donkey anti-Rabbit IgG- Alexa Fluor 488	ThermoFisher	Cat# A-21206; RRID: AB_2535792
Donkey anti-Rabbit IgG- Alexa Fluor 568	ThermoFisher	Cat# A-10042; RRID: AB_2534017
Donkey anti-Rabbit IgG- Alexa Fluor 647	ThermoFisher	Cat# A-31573; RRID: AB_2536183
Goat anti-Chicken IgY- Alexa Fluor 488	ThermoFisher	Cat# A-11039; RRID: AB_2534096
Biotinylated Goat Anti-Rabbit IgG Antibody	Vector Laboratories	Cat# BA-1000

**Bacterial and Virus Strains**		

AAV5-EF1a-DIO-eYFP-WPRE-hGH	Gift from Karl Deisseroth (unpublished)	Addgene Plasmid #27056; RRID: Addgene_27056
AAV5-EF1a-DIO-ChR2-eYFP-WPRE-hGH	Gift from Karl Deisseroth (unpublished)	Addgene Plasmid #20298; RRID: Addgene_20298
AAV5-EF1a-DIO-eNpHR3.0-eYFP-WPRE-hGH	Gradinaru et al., 2010	Addgene Plasmid #26966; RRID: Addgene_26966
AAV5-flex-taCasp3-TEVp	UNC, GTC vector core	Cat# 77699
AAV/DJ-CMV-mCherry-T2A-iCre	Vector Biolabs	Cat# VB7600
AAV/DJ-CMV-mCherry	Vector Biolabs	Cat# VB7777

**Biological Samples**		

Normal donkey serum (NDS)	Sigma-Aldrich	Cat# D9663
Bovine Serum Albumin (BSA)	Sigma-Aldrich	Cat# A2153

**Chemicals, Peptides, and Recombinant Proteins**		

Picric acid	Sigma-Aldrich	Cat# P6744
4% paraformaldehyde (PFA) in PBS	Santa Cruz Biotech.	Cat# CAS30525-89-4
Streptavidin conjugated to FITC	ThermoFisher	Cat# 11-4317-87
Streptavidin conjugated to DyLight 405	ThermoFisher	Cat# 21831
Streptavidin conjugated to Alexa Fluor 568	ThermoFisher	Cat# S11226
Streptavidin conjugated to Alexa Fluor 647	ThermoFisher	Cat# CS32357
Neurobiotin tracer	VectorLabs	Cat# SP-1120-50
Cesium methanesulfonate	Sigma-Aldrich	Cat# C1426-5G
Tetrodotoxin	Alomone labs	Cat# T-550
Charybdotoxin	Alomone labs	Cat# STC-325
Apamin	TOCRIS	Cat# 1652
Prolactin (Prl)	Prospec	Cat# CYT-1060
Ovine Prl	National Hormone and Peptide Program (NHPP)	Cat# AFP10692C
Sodium chloride	Sigma-Aldrich	Cat# S9888
Sodium bicarbonate	Sigma-Aldrich	Cat# S6297
D-(+)-Glucose	Sigma-Aldrich	Cat# G7528
Sodium phosphate monobasic dihydrate	Sigma-Aldrich	Cat# 71505
Potassium chloride	Sigma-Aldrich	Cat# P9333
Magnesium sulfate heptahydrate	Sigma-Aldrich	Cat# 63138
Calcium chloride dihydrate	Sigma-Aldrich	Cat# C5080
OCT Cryomount	Histolab	Cat# 45830
Triton X-100	Sigma-Aldrich	Cat# T8787
Sucrose	Sigma-Aldrich	Cat# S7903
Vectastain ABC kit	Vector Laboratories	Cat# PK-6100
3,3-Diaminobenzidine tetrahydrochloride hydrate (DAB)	Sigma-Aldrich	Cat# D5637
Phosphate buffer saline (PBS)	Santa Cruz Biotech.	Cat# SC-24946

**Critical Commerical Assays**		

Rat Prl ELISA kit	Sigma-Aldrich	Cat# RAB1153
Mouse Prl ELISA kit	ThermoFisher	Cat# EMPRL
Corticosterone competitive ELISA kit	ThermoFisher	Cat# EIACORT

**Experimental Models: Organisms/Strains**		

C57BL/6J mouse line	Charles River	N/A
Sprague-Dawley rat line (Crl:SD)	Charles River	N/A
DAT-Cre C57BL/6J mouse line	Ekstrand et al., 2007	N/A
Gal-Cre C57BL/6J mouse line	MMRRC_031060-UCD	N/A
tdTomato-floxed C57BL/6J mouse line	Jackson Laboratory 007914	N/A
Prlr-floxed C57BL/6 mouse line	Brown et al., 2016	N/A

**Software and Algorithms**		

Clampfit 10.2	MOLECULAR DEVICES	https://www.moleculardevices.com/
MATLAB 2019	MathWorks	https://www.mathworks.com/
Ethovision XT 11	Noldus	https://www.noldus.com/
BORIS	Friard and Gamba, 2016	http://www.boris.unito.it/
OriginPro 9	OriginLab	https://www.originlab.com/
ImageJ	NIH	https://imagej.nih.gov/ij/
Prism 8	GraphPad	https://www.graphpad.com/scientific-software/prism/
Illustrator CC 2019	Adobe Systems	https://www.adobe.com/
CorelDrawX8	CorelDRAW graphics suite	https://www.coreldraw.com/en/
Photoshop 2019	Adobe Systems	https://www.adobe.com/

### Resource Availability

#### Lead Contact

Requests for further information or reagents should be directed to and will be fulfilled by the Lead Contact, Christian Broberger (christian.broberger@dbb.su.se).

#### Materials Availability

This study did not generate new unique reagents.

#### Data and Code Availability

This study did not generate datasets or employ previously unreported custom computer code.

### Experimental Models and Subject Details

#### Animals

All animal experiments had received approval from the local ethical board, *Stockholms Norra Djurförsöksetiska Nämnd*, and were performed in accordance with the European Communities Council Directive of November 24, 1986 (86/609/EEC). Adult rodents were used, typically between 2 to 8 months old, according to the demands of each experiment (sexually inexperienced or parental state). Wild-type mice with C57BL/6J background were used, in addition to previously generated C57BL/6J *Slc6a*^Cre^ (DAT-Cre) knock-in (Ekstrand et al., 2007) and floxed-tdTomato mice (The Jackson Laboratory, strain datasheet – 007909). Gal-Cre BAC transgenic mice (strain: Tg(Gal-cre)KI87Gsat/Mmucd, citation ID: RRID:MMRRC_031060-UCD) were purchased from MMRRC. Wild-type Sprague Dawley (Crl:SD) rats were used (own breeding), and were originally acquired from Charles River. Animals were group-housed, up to five per cage, in a temperature (23°C) and humidity (55%) controlled environment, in a 12h light, 12h dark cycle with *ad libitum* access to food and water. Cages were changed on a weekly basis.

Prlr^lox/lox^ mice. Generation of Prlr^lox/lox^ animals has been previously described (Brown et al., 2016). Briefly, mice were generated on a C57BL/6 background, with exon 5 of the Prlr gene flanked by lox66 and lox71 sites, along with an inverted copy of green fluorescent protein (eGFP) reporter. Cre-mediated inversion of this sequence renders a non-functional Prlr (deletion of exons 5-10) and induction of eGFP expression under the control of the Prlr promoter. Thus, eGFP expression in the brain can be used as a marker of successful Prlr deletion (and as a marker of cells that normally express the Prlr). All mice were housed in individually ventilated cages with shredded paper nesting material and kept in temperature-controlled rooms (22 ± 1°C) on 12:12 hour light/dark cycles with *ad libitum* access to food and water. Males were initially group housed until paired with a female for the experiment.

### Method Details

#### Brain slice electrophysiology

Acute brain slices were prepared from mice and rats. Slices were cut on a vibratome (Leica VT1000S) to 250 μm thickness and continuously perfused with oxygenated aCSF containing (in millimolar): NaCl (127), KCl (2.0), NaH_2_PO_4_ (1.2), NaHCO_3_ (26), MgCl_2_ (1.3), CaCl_2_ (2.4), and D-glucose (10). Slices were exposed only to a single bath application of pharmacological compounds and were used for a single experiment. Whole-cell current- and voltage-clamp recordings were performed with micropipettes filled with intracellular solution containing (in millimolar), K-gluconate (140), KCl (10), HEPES (10), EGTA (10), and Na_2_ATP (2) or Cesium methanesulfonate (140), KCl (10), HEPES (10), EGTA (10), and Na_2_ATP (2) (pH 7.3 with KOH). Recordings were performed using a Multiclamp 700B amplifier, a DigiData 1440 digitizer, and pClamp 10.2 software (Molecular Devices). Slow and fast capacitative components were semi-automatically compensated. Access resistance was monitored throughout the experiments, and neurons in which the series resistance exceeded 15 MΩ or changed ≥ 20% were excluded from the statistics. Liquid junction potential was 16.4 mV and not compensated. The recorded current was sampled at 20 kHz. Spontaneous excitatory currents were sampled at the reversal of Cl^-^ (V_hold_ = −70 mV), and spontaneous inhibitory currents were sampled during short intervals – typically 1 min – at the reversal of fast excitatory neurotransmission (V_hold_ = +10 mV). All recordings were performed at near physiological temperature (33 ± 1°C). Reagents used in slice electrophysiology experiments; Prl (CYT-321 – PROSPEC), Neurobiotin™ tracer (Vector laboratories) was used in combination with Streptavidin, DyLight™ 405 conjugated (21831 Thermo Scientific) or Avidin-FITC (43-4411 Invitrogen). MATLAB and OriginPro8 were used for electrophysiological data analysis.

Identification of the Prl induced current was performed via the use of a voltage clamp ramp protocol, as illustrated in Figure 4L. The cell response to a one second duration voltage clamp ramp was recorded during baseline (or in the presence of a K^+^ channel blocker) – Ramp1, and following application of Prl – Ramp 2. Digital subtraction of the recorded currents (Ramp 2 – Ramp 1), led to the isolation of the Prl-induced current and its modulation by distinct K^+^ channel blockers. Prl was used at 200 nM, intracellular Cesium methanesulfonate at 140 mM, Charybdotoxin at 200 nM and Apamin at 300 nM.

#### Cell filling and reconstruction

Mouse and rat TIDA neurons and mouse *Gal*^+^ MPOA neurons were recorded in whole-cell mode with intracellular pipette solution as above, with the addition of 0.2% neurobiotin. After recording, slices were placed in fixative (4% paraformaldehyde/0.16% picric acid), washed in PBS, and incubated at 4°C for 72h in a solution containing FITC-conjugated avidin (1:2500, 43-4411, Zymed) and mouse anti-tyrosine hydroxylase immunoglobulin (1:2000, MAB318, Millipore). After extensive washing, slices were incubated in secondary Alexa-647-conjugated donkey-anti mouse antiserum (1:500, Invitrogen, A21202) at 4°C for 16h, washed again and mounted with 2.5% DABCO in glycerol. TIDA identity of all filled cells was confirmed with tyrosine hydroxylase (TH) immunoreactivity.

#### *In vitro* optogenetics

Photostimulation during slice whole-cell recordings was accomplished via a 3.4 W 594 nm LED mounted on the microscope oculars and delivered through the 63X objective’s lens. Photostimulation was controlled via the analog outputs of a DigiData 1440A, enabling control over the duration and intensity. The photostimulation diameter through the objective lens was ∼350 μm with illumination intensity typically scaled to 3 mW/mm^2^.

#### *In vivo* optogenetics

In optogenetic experiments, subjects were coupled via a ferrule patch cord to a ferrule on the head of the mouse using a zirconia split sleeve (Doric Lenses). The optical fiber was connected to a laser (CNI-MLL-III-635-200-5-LED, CNI lasers 200 mW) via a fiber-optic rotary joint (FRJ_1x1_FC-FC, Doric Lenses) to avoid twisting of the cable caused by the animal’s movement. After a testing session, DAT-Cre animals were uncoupled from the fiber-optic cable and returned to their home cage. The frequency and duration of photostimulation were controlled using custom-written LabView software. Laser power was controlled by dialing an analog knob on the power supply of the laser sources. Light power was measured from the tip of the ferrule in the patch cord before being installed in the brain (the ferrule-connector system) at different laser output settings, using an optical power and energy meter and a photodiode power sensor (Thorlabs). Upon identification of the fiber placement coordinates in brain tissue slides, irradiance (light intensity) was calculated using the brain tissue light transmission calculator based on (https://web.stanford.edu/group/dlab/cgi-bin/graph/chart.php) using laser power measured at the tip and the distance from the tip to the target brain region measured by histology. Animals showing no detectable viral expression in the target region and/or ectopic fiber placement were excluded from the analysis.

#### Immunohistochemistry

Mice were anesthetized with sodium pentobarbital (200 mg/kg, i.p., Sanofi-Aventis), then transcardially perfused with 10 mL Ca^2+^-free Tyrode’s solution (37°C) containing 0.2% heparin, followed by 10 mL fixative (4% paraformaldehyde and 0.4% picric acid in 0.16 M phosphate buffer (PBS), 37°C), then 50 mL ice-cold fixative. Whole brains were dissected, immersed in ice-cold fixative for 90 min then stored in 0.1M PBS (pH 7.4) containing 20% sucrose, 0.02% bacitracin, and 0.01% sodium azide for three days, before freezing with CO_2_. Coronal sections were cut at a thickness of 14 μm on a cryostat (Microm, Walldorf) and thaw-mounted onto gelatine-coated glass slides. For the dataset presented in Figures 6M–6W and S5 mice were deeply anesthetized with pentobarbital (100 mg/ kg^-1^) and brains collected following transcardial perfusion with 4% paraformaldehyde. Brains were post-fixed in the same fixative overnight then transferred to 30% sucrose solution for 48 hours prior to being frozen and stored at −80°C until processing. Three sets of 30-μm thick coronal sections through the forebrain were cut using a sliding microtome.

Immunohistochemistry for pSTAT5: Prior to immunofluorescence staining, antigen retrieval was performed by incubating sections for 15 minutes in citric acid (pH 7.4) at 80°C, then cooled at room temperature for a further 30 minutes. After a 1% H_2_O_2_ Tris-buffered wash, sections were incubated in rabbit pSTAT5 primary antibody (pSTAT5 Tyr 694, Cat#: C11C5, 1:500; Cell Signaling Technology) for 72 hours at 4°C. Primary antibody incubation was followed by Alexa 488-conjugated donkey anti-rabbit secondary antisera (1:500; Invitrogen). Immunofluorescence for tyrosine hydroxylase: Anti-TH antiserum was applied on sections at 4°C over-night (1:2000; raised in rabbit, AB152, Millipore). Primary antibody incubation was followed by Alexa 488-conjugated donkey anti-rabbit secondary antisera (1:500; Invitrogen). Immunofluorescence for NeuN: NeuN was detected with rabbit anti-NeuN primary antibody (1:500; Cell Signaling, D4G40). Primary antibody incubation was followed by Alexa-647-conjugated donkey anti-rabbit secondary antisera (1:500; Invitrogen). For the dataset presented in Figure 6N, pSTAT5 immunohistochemistry was performed using rabbit pSTAT5 primary antibody at 1:1000 dilution and visualized using DAB, as described previously (Brown et al., 2010). In data presented in Figure S5, immunohistochemistry for eGFP was performed according to Brown et al. (2016) using rabbit GFP primary antibody at 1:30,000 and visualized using DAB (Brown et al., 2016). Brightfield images were taken, and cell counts were made using ImageJ.

#### Confocal microscopy and cell counting

All brain slices were imaged by confocal microscopy (Zeiss, LSM 800) for subsequent analysis. Brain areas were determined according to their anatomy using Paxinos and Franklin Brain Atlas (Franklin and Paxinos, 2008). For arcuate nucleus and MPOA cell counts, the entire areas were cut, stained and counted. All counts were done manually using ImageJ software and blind to test conditions.

#### Carbon fiber microelectrodes

Carbon fiber working electrodes were produced by aspirating 7 μm diameter carbon fibers (Cytec engineered materials, Tempe, AZ) into borosilicate glass capillaries (1.2 mm O.D., 0.69 mm I.D., Sutter Instrument Co., Novato, CA). The capillaries were subsequently pulled with a commercial micropipette puller (Sutter Instrument, P-97) and sealed with epoxy (EpoTek 301, Epoxy Technology, Billerica, MA). The electrode tips were polished at a 45° angle on a diamond dust-embedded micropipette-bevelling wheel (Model BV-10, Sutter Instrument Co., Novato, CA). Electrodes were tested performing bath applications of known concentrations of dopamine. Only electrodes showing reaction kinetics typical of dopamine (as examined in current versus time plots, and current versus voltage plots) were used.

#### Fast-scan cyclic voltammetry

A Dagan Chem-Clamp potentiostat (Dagan Corporation, Minneapolis, MN) and two data acquisition boards (PCI-6221, National Instruments, Austin, TX) run by the TH 1.0 CV program (ESA, Chelmsford, MA) were used to collect all electrochemical data. Cyclic voltammograms were obtained by applying a triangular waveform potential (−0.4 to +1.3 V versus Ag/AgCl) repeated every 100 ms at a scan rate of 200 V/s (low pass Bessel filter at 3 kHz). Each cyclic voltammogram was a background-subtracted average of ten successive cyclic voltammograms taken at the maximum oxidation peak current. All electrodes were allowed to cycle for at least 15 min prior to recording to stabilize the background current. The recorded current response was converted to dopamine concentration via *in vitro* electrode calibration with standard dopamine solution after each experiment. Acquired data were analyzed and plotted using MATLAB routines and statistical analysis was performed using Prism 6.0 (GraphPad Software, La Jolla, CA).

#### Tail-tip whole blood sampling

Whole blood samples of 200 μL were collected from the tail vein of restrained mice and rats (Lee and Goosens, 2015). Only blood samples acquired within 2 min post-restraining were used for hormone measurements, and the subjects were then returned to their home cage. Briefly, the rodent’s tail was immersed for 30 s in 40°C water to dilate the tail vessels. Immediately after, a 23G needle was used to puncture the lateral tail vein, and whole blood was collected. Bleeding was stopped via applying gentle pressure to the tail at the level of the puncture with surgical cleaning tissue, and 2% chlorhexidine antiseptic solution was used for tail disinfection at the end of the procedure. Blood samples were refrigerated at 4°C for 30 min and then centrifuged at 4^ο^C at 2000 R*CF.* Following centrifugation, serum was collected and was frozen at −80°C for a maximal period of 2 months prior to performing ELISA measurements. All blood samples were acquired between 13:00 and 15:00 during the day.

#### Prolactin and corticosterone ELISA

96-well antibody pre-coated plates were used in a ready-to-use kits for both Prl (Sigma-Aldrich RAB1153 or ThermoFisher Scientific – Catalog number EMPrl), and corticosterone (ThermoFisher Scientific – Catalogue number EIACORT). A linear regression was used to fit the optical densities for the standard curve versus their concentration. The standard curve range for Prl was 30 to 20000 pg/mL. The standard curve range for corticosterone was 300 to 100000 pg/mL. Concentrations were calculated from the optical density at 450 nm of each sample. Appropriate sample dilutions were carried out to maintain detection in the linear part of the standard curve, and typically involved 1 to 3 for rat serum samples and 1 to 10 or 20 for mouse serum samples. Data acquired from the performed ELISAs are presented as absolute values. Differences between groups were identified by Student’s t test or ANOVA.

#### Viral vectors

For channelrhodopsin *in vitro* optogenetic studies, animals were injected in the arcuate nucleus with 200 nL of AAV5-EF1a-DIO-ChR2-eYFP-WPRE-hGH (Addgene20298) 8.41 × 10^12^ genomic copies per mL. For *in vivo* optogenetic experiments and neuron photoinhibition, 200 nL of AAV5-EF1a-DIO-eNpHR3.0-eYFP-WPRE-hGH (Addgene26966) 7.02 × 10^12^ genomic copies per mL were injected in the arcuate nucleus. Control groups were injected with 200 nL of AAV5-EF1a-DIO-eYFP-WPRE-hGH (Addgene27056) 5.82 × 10^12^ genomic copies per mL. Viral injections were performed bilaterally. Control injections of the Cre dependent viral constructs were performed in C57 wild-type mice; no fluorophore expression was detected in these animals. For targeted cell ablation of DAT^+^ arcuate nucleus neurons, animals were injected in the arcuate with 300 nL of AAV5-flex-taCasp3-TEVp 2.9 × 10^12^ genomic copies per mL. For deletion of Prlr from the MPOA, 1 μL of AAV/DJ-CMV-mCherry-T2A-iCre (Vector Biolabs VB7600) 1.4 × 10^13^ genomic copies per mL was injected into the MPOA. Control groups were injected with 1 μL of AAV/DJ-CMV-mCherry (Vector Biolabs VB7777) 3.7 × 10^13^ genomic copies per mL. Viral injections were performed bilaterally. Viruses were prepared by Vector BioLabs (Malvern, PA) and the University of Pennsylvania Vector Core.

#### Stereotactic surgery and viral gene transfer

Adult DAT-Cre male mice of 2-8 months age were stereotactically injected. Animals were anesthetized with isoflurane (1–5%) and placed in a stereotaxic frame (David Kopf Instruments). The virus was injected into the arcuate nucleus bilaterally using a pulled glass capillary (World Precision Instruments) by nanolitre pressure injection at a flow rate of 50 nL per min (Micro4 controller, World Precision Instruments; Nanojector II, Drummond Scientific). Stereotactic injection coordinates to target the arcuate nucleus were obtained from the Paxinos and Franklin atlas (Franklin and Paxinos, 2008) (Bregma: −1.8 mm, midline ± 0.1 mm, dorsal surface −5.5 mm). Ferrules and fiber-optic patch cords were purchased from Thorlabs and Doric Lenses, respectively. The virus-injected animals were housed individually during a three-day recovery period, and were then reintroduced to their breeding cage.

Adult male Prlr^lox/lox^ mice (8–10 wk old) were anesthetized under isoflurane and placed in a stereotaxic apparatus. Animals received bilateral 1 μL injections of either AAV/DJ-CMV-mCherry-T2A-iCre (AAV-Cre) or AAV/DJ-CMV-mCherry (control virus) into the MPOA. Coordinates were adjusted for weight of animal (20-25 g: +0.06 anterior to Bregma; −0.48 depth; 25-30 g: +0.07 anterior to Bregma, −0.49 depth). All injections were given at a rate of 100 nL/min, and the syringes were left *in situ* for 3 min before and 10 min after injections to allow diffusion of the virus. Animals were allowed to recover for three weeks before being used in the experiment.

#### Prolactin receptor knock-out in MPOA

To knock-out the Prlr gene from the MPOA, Prlr^lox/lox^ mice were injected with AAV-Cre. Successful Cre recombination results in the expression of eGFP and deletion of exons 5-10 of the Prlr gene. This leads to the complete absence of both short- and long-forms of the Prlr in the target tissue in the neurons that express the Cre recombinase (Brown et al., 2016). This design makes it possible to quantify the number of cells in which the Prlr is knocked-out via quantification of the eGFP fluorescence. Such information is provided in the present study in Figure S5.

Investigation of the functional readout of Prlr activation (through pSTAT5 immunohistochemistry), was assessed to verify that the receptor is not present. While pSTAT5 is not exclusively activated by Prl (other cytokines and hormones such as growth hormone can induce phosphorylation of STAT5 through their own receptors (Fleenor et al., 2006)), it is activated in all cells downstream of the long form of the Prlr (Ali and Ali, 1998; Brockman and Schuler, 2005; Buntin and Buntin, 2014; Cave et al., 2005; Gouilleux et al., 1994; Jaroenporn et al., 2009; Brown et al., 2010). In the present study, the lack of pSTAT5 signal following administration of Prl provides evidence of MPOA neurons being functionally unresponsive to Prl.

#### Behavioral tests

Rats or mice were acclimated to the testing facility for 1 h before testing. Behaviors were recorded using a digital video recording unit and scored using EthoVision (Noldus Information Technology) or BORIS (Friard and Gamba, 2016).

##### Parental behavior assay

We tested animals for parental behavior with their pups being typically 4-8 days old. The experiments presented in Figure 5, included parental behavior tested with the first, second and third litters. Testing was performed during the light phase and under 40 lux luminescence. Rodents were acclimated to the testing facility for 1 h before testing. The test subject (sire or dam), pups and nest were transferred to the testing arena with the other parent remaining in the home cage. The testing arena was an open field test arena, and fresh bedding was placed at the beginning of each trial. Trials were 1h in duration. To begin each assay, 2g of shredded cotton provided as nesting material was placed in eight pieces on one side of the arena and typically six pups were placed on the opposite side at maximal distance from each other and the nesting material (see Figure 5B). Immediately after the test subject was introduced, recordings of the exhibited behaviors were performed for 1h. Behavioral scoring included latency to first approach and total duration of approach/proximity to a pup, crouching over pups (by covering at least 50% of the pup’s body with the parent’s body), pup grooming, huddling over the pup, and pup retrieval (pick up the pup with the mouth and displace it from its original position). Total duration of nesting was also scored and nest quality was assessed at the end of the recording according to the following scale (Capone et al., 2005): 0, nesting material is scattered and proximal to its original placement; 1, few pieces of nesting material have been gathered in a flat platform but most pieces still scattered; 2, all nesting material has been gathered in a flat platform; 3, nesting material has been collected and a hooded nest has been constructed. Behaviors were recorded using a digital video recording unit and scored using EthoVision XT by Noldus.

Experiment presented in Figures 6M–6W. The experiment presented in Figures 6M–6W was performed at the University of Otago, using a pup exposure assay (Tsuneoka et al., 2015) during the dark cycle. All methods were approved by University of Otago Animal Ethics Committee. Briefly, males were paired with a wild-type female and co-housed together until day 3 post-partum (PP; the day of parturition was recorded as day 1 PP). On day 3 PP, males were removed from the home cage and singly housed in a novel cage for 24 hours. On the following day, males were transferred to a quiet testing room and allowed to acclimate for at least 15 minutes. Lids were replaced with clear plexiglass and a video camera placed directly above the cage to record behaviors. Following the acclimation period, four pups from the male’s original cage were placed in his cage opposite to his nest. Two of the control male’s litters had less than four pups, so extra pups were supplemented from other cages so that he was exposed to four pups during the test. Previous testing confirmed that paternal males will retrieve both their own or foreign pups at similar rates (unpublished obs). Paternal behaviors were recorded for 30 minutes. Following the end of the test, animals were injected with ovine Prl (5 mg/kg injection/i.p.), to assess the degree of Prl deletion following AAV-Cre administration, and perfused 45 minutes later.

Paternal behaviors were scored from the time all four pups were placed in the male’s cage. Latency to first approach pups, latency to retrieve all four pups, and duration of pup retrieval, proximity to pups, pup grooming, crouching over pups, and nesting were recorded using the program BORIS (Friard and Gamba, 2016). Animals which did not retrieve were assigned the value of the length of the test (1800 s). All behaviors were scored identically as the pup assay test used in Figures 5, 6, and 7, with the exception of nesting. In this case, because males already had a nest at the beginning of pup testing, nesting was considered as any adjustment or manipulation to the already existing nesting material.

##### Open field test (OFT)

OFTs were performed in a white acrylic glass box (45 × 45 × 40 cm) with an overhead lamp directed to the center of the field, providing 120 lux of illumination on the floor of the arena. Each mouse was placed in the corner of the apparatus and locomotion parameters were recorded for 15 min. All subjects went through a single 5 min session prior to the OFT day for acclimatization.

##### Elevated plus maze (EPM)

The EPM test was performed using a polyvinyl chloride maze comprising a central part (5x5 cm), two opposing open arms (32.5x5 cm), and two opposing closed arms (32.5x5x32.5 cm). The apparatus was set to a height of 50 cm, and the open arms were provided with uniform overhead illumination of 6 lux. Mice were placed in the open arm point close to the center facing the closed arms, and video recordings were performed for a total duration of 20 min. A day prior to the test, mice were placed in the arena for a period of 5 min for acclimatization.

##### Preference assay

Mice were placed in a custom three-chamber behavioral arena (70 × 35 × 25 cm acrylic glass) for 15 min. In the left chamber, an estrus female was introduced under a “cage” with bars that allow minimal interaction between the sire and the female. The middle compartment of the arena was empty, while the right compartment included a “cage” with the sire’s own pups. The time spent in each chamber was measured in control and eNpHR3 animals with photostimulation at 0.2 Hz.

##### Randomization and blinding

Behavioral data collection and analysis presented in Figures 6 and 7 were performed blind to experimental conditions. Anatomy data analysis but not tissue collection was blinded. Electrophysiological, voltammetry and behavioral data sampling and analysis presented in Figures 1, 2, 3, 4, and 5 were not blinded.

### Quantification and Statistical Analysis

No statistical methods were used to predetermine sample sizes but our sample sizes are similar to those reported in previous publications (Bendesky et al., 2017; Wu et al., 2014; Brown et al., 2017; Kohl et al., 2018). Data met the assumptions of the statistical tests used, and were tested for normality and equal variance. The ROUT method was used for identification of outliers (Motulsky and Brown, 2006). All t tests and one-way ANOVAs were performed using Graph Pad Prism software (Graphpad Software Inc.). The Tukey and Bonferroni *posthoc* tests were used as appropriate for one-way ANOVAs. Normality was determined by D’Agostino–Pearson normality test. Statistical significance was set at p < 0.05.
